# Assessing the robustness of clinical trials regarding novel therapies in inflammatory bowel disease

**DOI:** 10.1093/gastro/goag051

**Published:** 2026-07-16

**Authors:** Jieqi Zheng, Pinwei Huang, Li Li, Shanshan Huang, Rirong Chen, Shenghong Zhang

**Affiliations:** Department of Gastroenterology, The First Affiliated Hospital, Sun Yat-sen University, Guangzhou, 510080, P.R. China; Department of Gastroenterology, The First Affiliated Hospital, Sun Yat-sen University, Guangzhou, 510080, P.R. China; Zhongshan School of Medicine, Sun Yat-sen University, Guangzhou, 510080, P.R. China; Department of Gastroenterology, The First Affiliated Hospital, Sun Yat-sen University, Guangzhou, 510080, P.R. China; Department of Gastroenterology, The First Affiliated Hospital, Sun Yat-sen University, Guangzhou, 510080, P.R. China; IBD Center and Zane Cohen Centre for Digestive Diseases, Lunenfeld Tanenbaum Research Institute, Mount Sinai Hospital, Toronto, Ontario, M5G 1X5, Canada; Guangxi Hospital Division of The First Affiliated Hospital, Sun Yat-sen University, Nanning, 530022, P. R. China

**Keywords:** inflammatory bowel disease, fragility index (FI), novel therapy, randomized clinical trial

## Abstract

**Background:**

Increasing randomized clinical trials evaluating novel therapies for inflammatory bowel disease necessitated the scrutiny of statistical robustness. This study aimed to quantify their fragility and identify factors associated with robustness.

**Methods:**

This cross-sectional analysis included randomized clinical trials studying biologics, small-molecule inhibitors, fecal microbiota transplantation (FMT), and stem cell therapy (SCT), and then the calculated fragility index (FI) and continuous fragility index (CFI) for binary and continuous outcomes, respectively. Factors affecting robustness were analysed through correlation analysis and multiple linear regression.

**Results:**

Among 129 trials from 53 studies, the median FI and CFI were 6 and 14.8, respectively. The FI varied significantly by the treatment type, trial phase, outcome type, and *P* values. The FI was positively correlated with the sample size (*ρ* = 0.734, *P *< 0.001), discontinuations (*ρ* = 0.479, *P *< 0.001), publication year (*ρ* = 0.253, *P *= 0.017), impact factor (*ρ* = 0.368, *P *< 0.001), and events percentage (*ρ* = 0.299, *P *= 0.005). The CFI was influenced by the outcome type and was strongly correlated with the sample size. After adjustment for other characteristics, biologics/small-molecule drugs displayed enhanced robustness relative to FMT/SCT (correlation coefficient *B* with the natural logarithm of FI: *B *= 0.283, *P *= 0.011). The primary or co-primary outcome exhibited greater robustness than did the other outcomes (FI: *B *= 0.288, *P *= 0.013; CFI: *B *= 0.459, *P *= 0.024). The sample size was positively correlated with both the FI (*B *= 0.001, *P *= 0.021) and the CFI (*B *= 0.001, *P *= 0.019), whereas discontinuation did not significantly affect the robustness.

**Conclusion:**

Randomized clinical trials of novel inflammatory bowel disease therapies exhibit varying robustness and are influenced by multiple study characteristics. Trials of biologics or small molecules, those with positive primary outcomes, and larger studies demonstrated greater robustness, supporting that robustness should be considered in future research when interpreting the efficacy.

## Introduction

Inflammatory bowel disease (IBD) is a nonspecific, chronic, and relapsing gastrointestinal inflammatory disorder that can be divided into Crohn’s disease and ulcerative colitis [1]. Its pathogenesis involves interactions between multiple factors, including genetics, impaired intestinal mucosal barrier, dysbiosis of gut microbiota, and environmental exposures [2]. Traditional therapies for IBD include corticosteroids, aminosalicylates, and immunomodulators such as thiopurines [3, 4]. With an enhanced understanding of the pathogenic mechanisms of IBD, an increasing number of novel treatments have emerged, such as biologics, small-molecule inhibitors, fecal microbiota transplantation (FMT), and stem cell therapy (SCT), which have offered new options for patients with inadequate response to conventional treatments [5–8].

Over the past decade, numerous randomized clinical trials (RCTs) have evaluated novel therapies for IBD. However, these trials relied solely on the *P* value to determine treatment efficacy, which is not comprehensive enough and may have affected the reliability of their results [9]. Specifically, an overreliance on the *P* value can easily lead to the results being reversed, as the strength of rejecting the null hypothesis is weak when the *P* value approaches 0.05 [10]. Therefore, Walsh *et al.* proposed the fragility index (FI) in 2014 to measure the stability of results. The FI represents the minimum number of events required to shift a statistical result from significant to non-significant, achieved by the sequential movement of one event at a time [11]. The fragility quotient (FQ), continuous fragility index (CFI), and continuous fragility quotient (CFQ) are derivative concepts of the FI. A higher FI usually signifies more robust evidence of therapeutic efficacy, reflecting greater reliability of the conclusions of the trial. It makes up for the deficiency of the *P* value in quantifying the robustness of conclusions against data perturbations by providing an innovative solution for assessing the stability of the trial results.

Despite the increasing number of clinical trials on novel therapies for IBD, there remains a paucity of comprehensive assessments of the robustness of their findings. To address this knowledge gap, we conducted a comprehensive review of novel therapeutic trials that have been initiated over the past decade. By employing the FI and its derived indices, we performed a multidimensional assessment of the robustness of the trial results and further investigated the potential determinants influencing robustness. This study seeks to provide critical insights into the evidentiary reliability of emerging therapeutic interventions for IBD, with the ultimate objective of informing the design of future clinical trials and therapeutic decision-making in clinical practice.

## Methods

### Study design

To identify relevant RCTs, we searched PubMed and Web of Science from 1 January 2014 to 10 November 2024, focusing on RCTs related to novel IBD therapies (defined as biologics, small-molecule inhibitors, FMT, and SCT) [6, 12]. The search strategy targeted key terms, including tumor necrosis factor-α, interleukins, integrin, sphingosine-1-phosphate receptor, phosphodiesterase 4, Janus kinase, FMT, and SCT. The search strategy is shown in Supplementary Table S1. Additional RCTs were obtained from the reference lists of relevant reviews or meta-analyses.

### Eligibility criteria

Studies that met the following criteria were included: (i) participants were diagnosed with IBD; (ii) interventions involved any novel IBD therapies; (iii) control measures included traditional treatments such as corticosteroids, immunosuppressants, and 5-aminosalicylic acid, or a placebo; (iv) at least one statistically significant result was reported, with the *P* value recalculated using the two-sided Fisher’s exact test (binary outcomes) or Welch *t*-test (continuous outcomes) being <0.05; and (v) clinical trials utilized an equal randomization design, such as 1:1 or 1:1:1.

Studies were exclude if they met any of the flowing criteria: (i) did not evaluate the efficacy of the novel therapies, where efficacy was defined as clinical, endoscopic, or histological remission or response, or changes in activity scores (e.g. Mayo score, Crohn’s Disease Activity Index, Harvey–Bradshaw Index, and Simple Endoscopic Score for Crohn’s Disease) or inflammatory markers (e.g. C-reactive protein, fecal calprotectin, and erythrocyte sedimentation rate); (ii) were not published in English; (iii) lacked the data required to calculate the FI or CFI; or (iv) were secondary publications of an already-included RCT (e.g. standalone post-hoc or subgroup analyses), to prevent duplication and maintain data independence.

When multiple outcomes met the criteria, the primary outcome was prioritized, followed by secondary outcomes and, finally, other outcomes. For studies with both induction and maintenance phases, each phase was considered as a separate trial. Multi-arm studies were split into multiple two-arm comparisons.

### Data extraction

For the included studies, the following data were systematically extracted: first author’s name, year of publication, journal title, impact factor, type of disease, study design (multi- or single-center), trial phase, blinding method, analysis type, intervention and control measures, outcome metrics (including definitions and timing of assessments), follow-up duration, reported *P* value, sample size, and number of early withdrawals. For studies reporting binary outcomes, the number of events in the intervention and control groups was recorded, whereas, for continuous outcomes, the means and standard deviations were extracted for each arm. When standard deviations were unavailable, they were derived from the standard error, 95% confidence interval (CI), range, or interquartile range (IQR) [13, 14].

### FI calculations

The robustness of efficacy outcomes in the novel therapy trials was mainly measured by using the FI. This process involved repeatedly converting one “non-event” into an “event” within the group, recalculating the *P* value at each step, until the result lost statistical significance. The total number of repetitions corresponds to the FI. A lower FI value indicates that the trial results are less stable and more susceptible to minor fluctuations. FI calculations were performed by using an online tool available at https://clincalc.com/Stats/FragilityIndex.aspx [11].

Additionally, the CFI, FQ, and CFQ were calculated as supplements. The CFI was computed by using an iterative algorithm. First, datasets representing continuous outcomes for both the intervention and control groups were input, followed by Welch’s *t*-test. If the *P* value was <0.05, then the data point closest to the group mean in the dataset with the higher mean was transferred to the other group. This process was repeated until the *P* value exceeded 0.05, with the total number of data points moved reflecting the CFI. Because of difficulties in obtaining the original datasets, simulated datasets were generated by using normally distributed random numbers based on the sample sizes, means, and standard deviations of the experimental and control groups. To enhance the stability of the estimates, this process was executed over five iterations and the mean CFI was calculated. These calculations were performed by using the web application https://jmcaldwell.shinyapps.io/CFIApp/ [15]. The FQ was calculated by dividing the FI by the sample size, which could reduce the impact of the sample size on the stability of the binary outcome studies [16]. For instance, an FQ of 0.05 means that changing the results of only 5% of the patients in the total sample can make the originally significant results non-significant. Similarly, the CFQ was calculated by dividing the CFI by the sample size.

### Statistical analysis

Continuous variables are described using the median (IQR) and categorical variables are presented as counts (percentages). Histograms were used to visualize the distributions of the FI, CFI, FQ, and CFQ.

To investigate the correlation between outcome stability and study characteristics, the Wilcoxon rank-sum test and the Kruskal–Wallis *H* test were employed for dichotomous and multicategorical variables, respectively. *P* values of <0.05 were considered statistically significant. Additionally, Spearman’s rank correlation analysis was used to calculate the correlation coefficients between continuous study characteristics and stability. Correlation coefficients of ≥0.8, 0.6–0.8, 0.4–0.6, and < 0.4 were considered very strong, strong, moderate, and weak correlations, respectively. A sensitivity analysis was conducted by including studies that lost statistical significance upon *P*-value recalculation with an imputed FI value of 0 to test the stability of the findings. Data analysis was performed by using IBM SPSS Statistics 25.

Multiple linear regression was used to further explore the impact of the study characteristics on the study stability. All study characteristics potentially associated with the FI in the preceding correlation analysis were incorporated into the regression model. To mitigate the effects of multicollinearity, variables with highly correlated characteristics (variance inflation factors of >10) were excluded from the analysis. Additionally, the induction/maintenance period was only distinguished in studies on biologics or small-molecule drugs and specific *P* values were reported in limited studies; therefore, these characteristics were also removed from consideration. Given that the type of analysis affected the number of events and sample size for calculation, it was included in the linear model. Before constructing the regression model, logarithmic transformations of FI and CFI were applied if the data did not follow a normal distribution. The unstandardized coefficient *B* and its corresponding 95% CI were calculated to determine the strength of the relationship. The *R*-squared statistic was utilized to measure the goodness of fit of the model, with a value of >0.3 deemed as acceptable.

## Results

### Study characteristics

**Figure 1:**
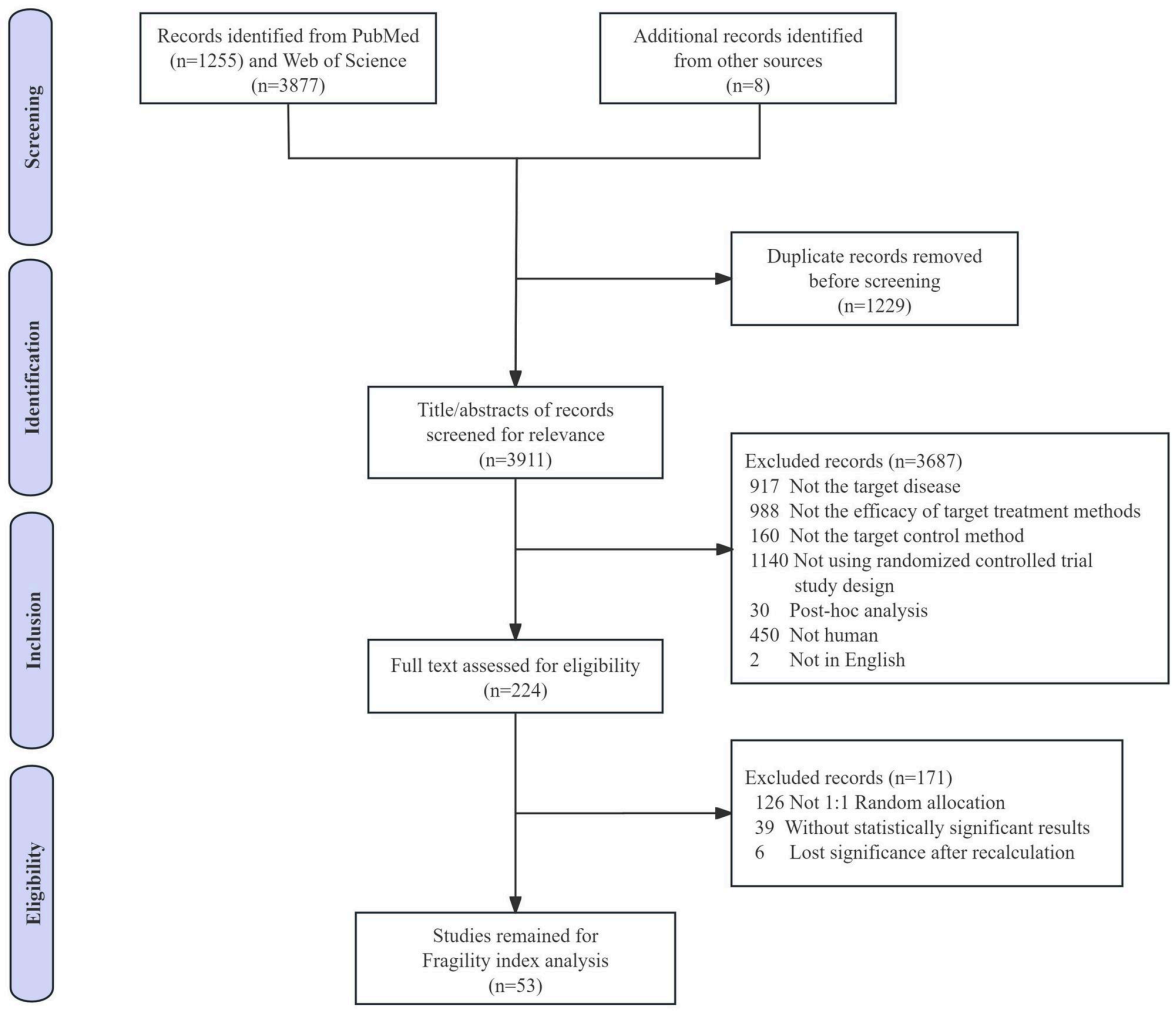
Flowchart of the study selection.

A total of 5,140 studies were screened, with 1,255 identified from PubMed, 3,877 from Web of Science, and 8 traced from references. Following the removal of duplicates, 3,911 studies were screened by the title and abstract. After the exclusion of ineligible studies, 224 full texts were assessed and exclusions were made for the following reasons: non-1:1 randomization (*n* = 126), lack of statistically significant outcomes (*n* = 39), and loss of significance after recalculations (*n* = 6). Finally, 53 studies were analysed (Fig. 1). Detailed information regarding the six studies excluded due to loss of significance after recalculations is summarized in Supplementary Table S2.

**Figure 2:**
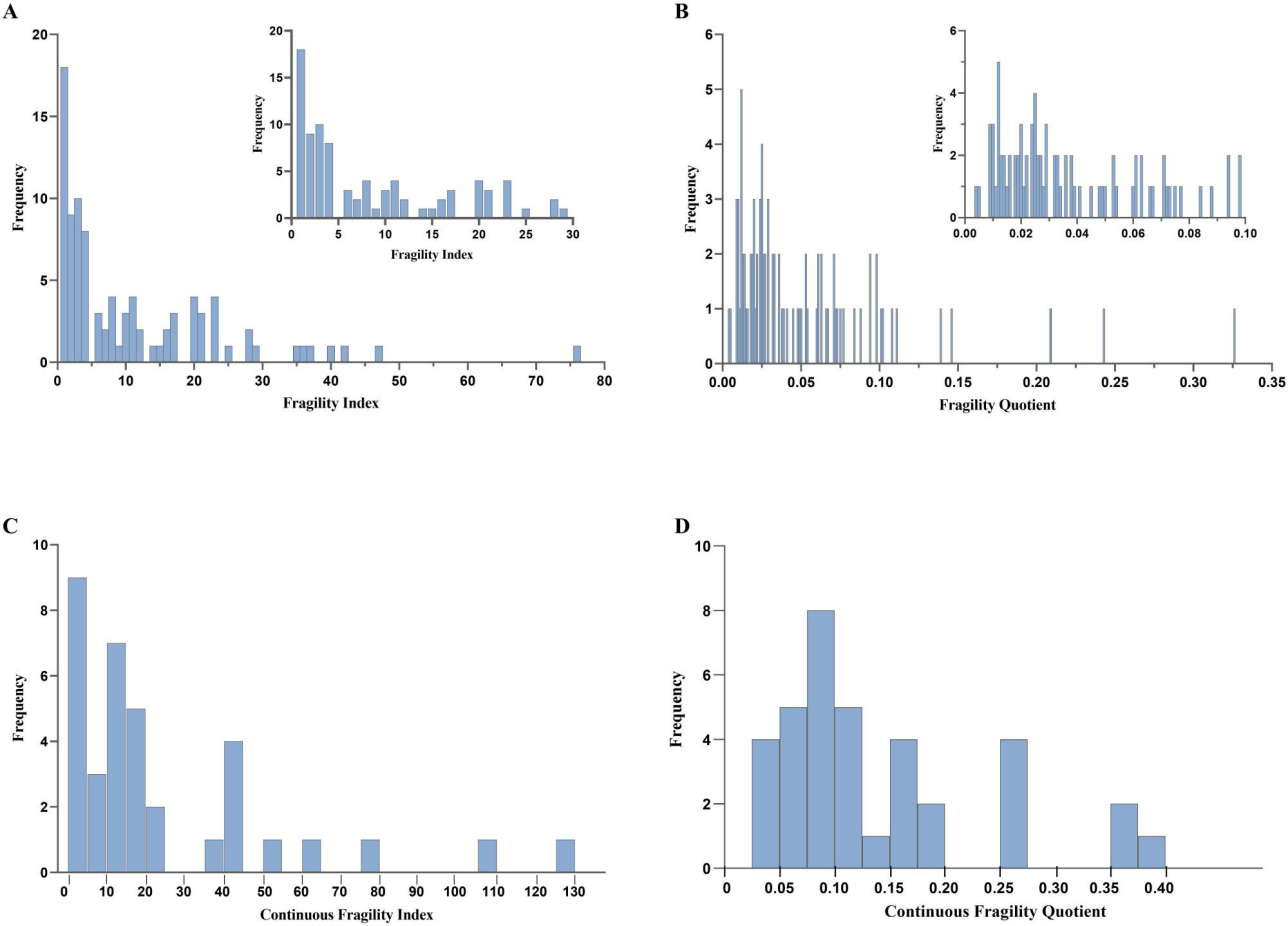
Distribution of the FI, FQ, CFI, and CFQ. (A) FI, (B) FQ, (C) CFI, (D) CFQ.

The characteristics of the 129 trials extracted from the 53 studies are summarized in Table 1. Among these trials, 93 were used for calculation of the FI and 36 were used for the CFI. Of the 53 studies, 20 (37.7%) and 33 (62.3%) focused on Crohn’s disease and ulcerative colitis, respectively. Additionally, 6 (11.3%) studies investigated FMT, 7 (13.2%) examined SCT, 25 (28.3%) involved biologics, and 15 (47.2%) pertained to small-molecule drugs. The frequency distribution diagrams of the FI, CFI, FQ, and CFQ are shown in Fig. 2. Comprehensive information on the study characteristics and calculated data for each included study are provided in Supplementary Tables S3–S6.

**Table 1:** Description of included studies.

Characteristics	All studies (*N *= 53)	Study for FI and FQ (*N *= 47)	Study for CFI and CFQ (*N *= 16)
Number of trials	129	93	36
Disease			
Crohn’s disease	20 (37.7)	16 (34.0)	7 (43.8)
Ulcerative colitis	33 (62.3)	31 (66.0)	9 (56.3)
Treatment			
FMT	6 (11.3)	6 (12.8)	1 (6.3)
SCT	7 (13.2)	4 (8.5)	3 (18.8)
Biologics	25 (28.3)	24 (51.1)	5 (31.3)
Small-molecule drugs	15 (47.2)	13 (27.7)	7 (43.8)
Year of publication, median (IQR)	2019 (2016–2022)	2019 (2016–2022)	2019 (2017–2020)
Impact factor, median (IQR)	29.4 (12.6–158.5)	29.4 (12.6–158.5)	29.4 (13.35–101.79)
Trial phase			
2	22 (41.5)	19 (40.4)	9 (56.3)
3	23 (43.4)	22 (46.8)	4 (25.0)
1/2	3 (5.7)	2 (4.3)	1 (6.3)
2/3	1 (1.9)	1 (2.1)	1 (6.3)
Not mentioned	4 (7.5)	3 (6.4)	1 (6.3)
Trial period			
Induction	25 (63.5)	22 (59.5)	9 (75.0)
Maintenance	13 (32.5)	13 (35.1)	3 (25.0)
Both	2 (5.0)	2 (5.4)	0 (0.0)
Type of analysis			
Intention-to-treat	33 (62.3)	29 (61.7)	11 (68.8)
Modified intention-to-treat	15 (28.3)	14 (29.8)	4 (25.0)
Per-protocol	3 (5.7)	2 (4.3)	1 (6.3)
Modified intention-to-treat and per-protocol	1 (1.9)	1 (2.2)	0 (0.0)
All	1 (1.9)	1 (2.2)	0 (0.0)
Blinding type			
Double-blind	49 (92.5)	46 (97.9)	13 (81.3)
Open-labeled	4 (7.5)	1 (2.1)	3 (18.8)
Center			
Multicenter	46 (86.8)	42 (89.4)	13 (81.3)
Single-center	7 (13.2)	5 (10.6)	3 (18.8)
Outcome type			
Primary	76 (58.9)	68 (73.1)	8 (12.5)
Co-primary	10 (7.8)	10 (10.8)	0 (0.0)
Secondary	27 (20.9)	14 (15.1)	13 (20.2)
Others	16 (12.4)	1 (1.1)	15 (67.3)
Outcome definition			
Clinical	85 (65.9)	64 (68.8)	21 (58.3)
Endoscopic	18 (14.0)	13 (14.0)	5 (13.9)
Others	10 (7.8)	0 (0.0)	10 (27.8)
Composite	16 (12.4)	16 (17.2)	0 (0.0)
Sample size, median (IQR)			
Total	135 (82–333.5)	194 (82–340)	103 (64.5–135.5)
Intervention	68 (41–168.5)	99 (41–170)	52 (32.8–68.5)
Control	67 (41–165)	96 (41–170)	50 (31.8–67.0)
Number of discontinuations, median (IQR)			
Total	17 (8–33)	23 (9–38.75)	11 (3–17)
Intervention	6 (3–13)	8 (4–18.5)	3.5 (1.8–5)
Control	10 (4–20)	12 (5–23.5)	4 (1–7.8)
Number of events, median (IQR)			
Total	–	67 (21–102)	–
Intervention	–	49 (15–66.5)	–
Control	–	18 (6–36)	–
Standard deviation, median (IQR)			
Intervention	–	–	3.4 (2.1–60.6)
Control	–	–	3.4 (2.1–78.5)
Reported *P* value			
<0.05–0.01	45 (34.9)	34 (36.6)	11 (30.55)
<0.01–0.001	24 (18.6)	17 (18.3)	7 (19.45)
<0.001	45 (34.9)	38 (30.9)	7 (19.45)
Not reported	15 (11.6)	4 (4.3)	11 (30.55)
Recalculated *P* value			
<0.05–0.01	–	35 (37.6)	–
<0.01–0.001	–	23 (24.7)	–
<0.001	–	35 (37.6)	–

Data are presented as counts *N* (%) or medians (IQR). Each study was divided into multiple trials according to the different periods, groups, and outcomes. The data represented by the dash indicates not applicable.

### Correlation between FI and study characteristics

The results of the correlation analysis are summarized in Table 2. The median FI was 6 and the median CFI was 14.8. Treatment type was significantly correlated with the FI (*P *= 0.001). Specifically, biologics (median [IQR]: 10 [3, 20]) and small-molecule drugs (median [IQR]: 8 [1, 25.5]) exhibited higher FIs than did FMT (median [IQR]: 1.5 [1, 2]) and SCT (median [IQR]: 2.5 [1.75, 4]). The trial phase was also significantly associated with the FI (*P *< 0.001), with phase 3 trials demonstrating relatively higher FIs (median [IQR]: 11 [4, 23]). Additionally, higher FIs were observed for co-primary and clinical outcomes with medians (IQRs) of 20 (9.75, 23) and 8 (3, 19.25), respectively. For both the reported and recalculated *P* values, a consistent trend was observed in which smaller *P* values were associated with larger FI values (*P *< 0.001).

**Table 2:** Associations between trial characteristics and FI.

Trial characteristics	FI			FQ		CFI			CFQ	
Categorical variable	Trials for FI and FQ (%)	Median FI (IQR)	*P* value	Median FQ (IQR)	*P* value	Trials for CFI and CFQ (%)	Median CFI (IQR)	*P* value	Median CFQ (IQR)	*P* value
Total	93 (100.0)	6 (2, 17)	–	0.0317 (0.0183, 0.0648)	–	36 (100.0)	14.8 (5.7, 39.3)	–	0.1017 (0.0753, 0.1917)	–
Disease										
Crohn’s disease	42 (45.2)	6.5 (2, 20)	0.759	0.0304 (0.0171, 0.0601)	0.223	13 (36.1)	12.2 (3.9, 16.2)	0.078	0.1017 (0.0919, 0.1759)	0.729
Ulcerative colitis	51 (54.8)	6 (2, 16)		0.0202 (0.0196, 0.0732)		23 (63.9)	16.4 (6.6, 43.8)		0.1010 (0.0725, 0.2000)	
Treatment										
FMT	8 (98.6)	1.5 (1, 2)	0.001	0.0280 (0.0145, 0.0376)	0.127	2 (5.6)	12.8 (10.0, 16.0)^a^	0.087	0.2099 (0.1607, 0.2590)^a^	0.597
Stem cell	10 (10.8)	2.5 (1.75, 4)		0.0186 (0.0102, 0.0329)		6 (16.7)	3.9 (2.2, 21.0)		0.0929 (0.0601, 0.2561)	
Biologics	50 (53.8)	10 (3, 20)		0.0304 (0.0213, 0.0681)		17 (47.2)	16.8 (12.7, 57.6)		0.1010 (0.0814, 0.1344)	
Small-molecule drugs	25 (26.9)	8 (1, 25.5)		0.0488 (0.0131, 0.0912)		11 (30.6)	12.0 (4.8, 42.6)		0.1644 (0.0516, 0.3610)	
Trial phase										
2	28 (30.1)	2 (1, 3)	<0.001	0.0224 (0.0122, 0.0402)	0.083	17 (47.2)	13.2 (7.6, 17.8)	0.456	0.1138 (0.0784, 0.1759)	0.107
3	55 (59.1)	11 (4, 23)		0.0340 (0.0202, 0.0670)		14 (38.9)	26.4 (4.0, 64.8)		0.0919 (0.0721, 0.1326)	
1/2	3 (3.2)	6.3 (1, 17)^a^		0.1032 (0.0323, 0.2429)^a^		2 (5.6)	21.4 (20.6, 22.2)^a^		0.2610 (0.2512, 0.2707)^a^	
2/3	1 (1.1)	2 (–)^b^		0.0328 (–)^b^		2 (5.6)	12.8 (9.8, 15.8)^a^		0.2099 (0.1607, 0.2590)^a^	
Not mentioned	6 (6.5)	4 (1.75, 6.5)		0.0499 (0.0330, 0.0793)		1 (2.8)	1.0 (–)^b^		0.0455 (–)^b^	
Trial period										
Induction	50 (66.7)	8 (2.75, 17.75)	0.035	0.0296 (0.0144, 0.0624)	0.038	22 (78.6)	17.4 (11.2, 46.5)	0.259	0.1014 (0.0739, 0.1646)	0.823
Maintenance	25 (33.3)	11 (6, 25.5)		0.0569 (0.0257, 0.0985)		6 (21.4)	14.7 (4.2, 16.5)		0.1308 (0.0667, 0.2027)	
Type of analysis										
Intention-to-treat	61 (65.6)	8 (2.5, 18.5)	0.141	0.0317 (0.0209, 0.0711)	0.271	25 (69.4)	16.4 (5.7, 43.6)	0.056	0.1217 (0.0670, 0.2551)	0.501
Modified intention-to-treat	27 (29.0)	3 (1, 20)		0.0325 (0.0189, 0.0599)		8 (22.2)	13.7 (12.1, 16.3)		0.1078 (0.0972, 0.1394)	
Per-protocol	5 (5.4)	3 (1.5, 10)		0.0155 (0.0094, 0.1303)		3 (8.3)	3.47 (2.6, 4.0)^a^		0.0836 (0.0650, 0.0952)^a^	
Blinding type										
Double-blind	90 (96.8)	6.5 (2, 17.75)	0.156	0.0306 (0.0184, 0.0663)	0.910	30 (83.3)	15.2 (9.5, 43.0)	0.062	0.1078 (0.0758, 0.1721)	0.671
Open-labeled	3 (3.2)	2 (1, 4)^a^		0.0392 (0.0179, 0.0606)^a^		6 (16.7)	3.9 (2.2, 21.0)		0.0929 (0.0601, 0.2561)	
Center										
Multicenter	85 (91.4)	7 (2, 20)	0.349	0.0286 (0.0168, 0.0632)	0.081	31 (86.1)	14.2 (4.8, 42.8)	0.731	0.0985 (0.0744, 0.1653)	0.192
Single-center	8 (8.6)	4 (2, 7.5)		0.0499 (0.0341, 0.0915)		5 (13.9)	18.2 (7.6, 23.3)		0.2512 (0.1031, 0.2649)	
Outcome type										
Primary	68 (73.1)	6.5 (2, 17)	0.002	0.0343 (0.0209, 0.0728)	0.002	8 (22.2)	15.5 (12.1, 42.8)	0.007	0.1091 (0.0942, 0.3638)	0.267
Co-primary	10 (15.1)	20 (9.75, 23)		0.0529 (0.0317, 0.0675)		0 (0.0)	–		–	
Secondary	14 (10.8)	2.5 (1, 5)		0.0183 (0.0122, 0.0270)		13 (36.1)	8.6 (3.2, 14.3)		0.0952 (0.0583, 0.1558)	
Others	1 (1.1)	1 (–)^b^		0.0085 (–)^b^		15 (41.7)	22.2 (9.8, 60.6)		0.1217 (0.0744, 0.2512)	
Outcome definition										
Clinical	64 (68.8)	8 (3, 19.25)	0.011	0.0353 (0.0227, 0.0728)	0.008	21 (58.3)	13.2 (4.8, 18.2)	0.255	0.1017 (0.0784, 0.1759)	0.265
Endoscopic	13 (14.0)	3 (1, 23)		0.0411 (0.0164, 0.0689)		5 (13.9)	42.6 (9.7, 43.1)		0.361 (0.0792, 0.3701)	
Others	0 (0.0)	–		–		10 (27.8)	25.9 (8.6, 56.1)		0.0938 (0.0712, 0.1616)	
Composite	16 (17.2)	2 (1.25, 3.75)		0.0178 (0.0126, 0.0290)		0 (0.0)	–		–	
Reported *P* value										
<0.05–0.01	34 (36.6)	1.5 (1, 3)	<0.001	0.0147 (0.0118, 0.0246)	<0.001	11 (30.6)	12.2 (3.8, 14.2)	0.126	0.1017 (0.0905, 0.1683)	0.602
<0.01–0.001	17 (18.3)	6 (3, 19)		0.0270 (0.0222, 0.0392)		7 (19.4)	16.8 (4.8, 42.6)		0.1138 (0.0933, 0.3610)	
<0.001	38 (40.9)	20 (11.75, 28)		0.0707 (0.0503, 0.0976)		7 (19.4)	9.8 (6.6, 20.0)		0.1471 (0.0725, 0.2590)	
Not reported	4 (4.3)	3.5 (1.5, 4)		0.0289 (0.0127, 0.0558)		11 (30.6)	43.8 (4.8, 77.4)		0.0865 (0.0615, 0.1653)	
Recalculated *P* value										
<0.05–0.01	35 (37.6)	1 (1, 3)	<0.001	0.0155 (0.0105, 0.0215)	<0.001	–	–	–	–	–
<0.01–0.001	23 (24.7)	7 (3, 10)		0.0317 (0.0244, 0.0411)		–	–		–	
<0.001	35 (37.6)	21 (16, 28)		0.0717 (0.0529, 0.1019)		–	–		–	
Continuous variable		Correlation coefficient	*P* value	Correlation coefficient	*P* value		Correlation coefficient	*P* value	Correlation coefficient	*P* value
Year of publication		0.253	0.017	0.257	0.015		0.162	0.345	0.071	0.682
Impact factor		0.368	<0.001	0.053	0.624		0.426	0.010	0.050	0.770
Sample size										
Total		0.734	<0.001	0.246	0.021		0.757	<0.001	0.133	0.438
Intervention		0.726	<0.001	0.233	0.029		0.756	<0.001	0.129	0.452
Control		0.741	<0.001	0.256	0.016		0.753	<0.001	0.130	0.451
Number of discontinuations										
Total		0.479	<0.001	0.191	0.076		−0.130	0.465	−0.228	0.195
Intervention		0.394	<0.001	0.125	0.250		0.103	0.563	−0.146	0.411
Control		0.504	<0.001	0.212	0.049		0.058	0.742	−0.180	0.309
Percentage of discontinuation		0.025	0.813	0.100	0.334		−0.609	<0.001	−0.286	0.101
Number of events										
Total		0.693	<0.001	0.317	0.003		–	–	–	–
Intervention		0.795	<0.001	0.427	<0.001		–	–	–	–
Control		0.506	<0.001	0.127	0.240		–	–	–	–
Percentage of events		0.299	0.005	0.289	0.006		–	–	–	–
Standard deviation										
Intervention	–	–	–	–	–		0.161	0.348	−0.123	0.476
Control	–	–	–	–	–		0.140	0.415	−0.094	0.584

Assessed by using Wilcoxon rank-sum tests for binary variables, Kruskal–Wallis *H* tests for multiple categorical variables, and Spearman’s correlation coefficient for continuous variables. The data represented by the dash indicates not applicable.

a Presented as mean (minimum maximum), as there are fewer than four data points.

b Presented as unique data. Percentage of discontinuation, total discontinuation divided by total sample size. Percentage of events, total number of events divided by total sample size.

For continuous variables, total sample size (*ρ* = 0.734, *P *< 0.001) and total number of premature discontinuations (*ρ* = 0.479, *P *< 0.001) exhibited strong and moderate positive correlations with FIs, respectively, while publication year, impact factor, and percentage of events showed weak positive correlations with FIs.

The study characteristics associated with the FQ were largely consistent with those related to the FI, with the exception of treatment type, trial phase, journal impact factor, and total discontinuation number, which did not exhibit statistically significant relationships with the FQ. The sensitivity analysis that included studies with an imputed FI value of 0 yielded results that were consistent with those of the primary analysis, demonstrating preserved direction and magnitude for the correlations of the FI and FQ with study characteristics, respectively (Supplementary Table S7).

A significant difference in CFIs was observed across outcome types (*P *= 0.007). A trend toward higher CFIs was observed for biologics or small-molecule drugs (median [IQR]: 15.2 [9.5, 43.3]) compared with FMT or SCT (median [IQR]: 6.9 [2.9, 19.4]), though the difference was not statistically significant (*P *= 0.070). Moreover, the CFI demonstrated a moderate correlation with the journal impact factor (*ρ* = 0.426, *P *= 0.010) and a strong correlation with the sample size (*ρ* = 0.757, *P *< 0.001). Conversely, the CFI was strongly negatively correlated with the discontinuation percentage (*ρ* = −0.609, *P *< 0.001). None of the characteristics showed a statistically significant association with the CFQ.

### Multiple linear regression analysis of factors affecting FI

Multiple linear regression analysis was performed to determine the factors that significantly contributed to trial robustness. The treatment type, trial phase, outcome type, analysis type, year of publication, impact factor, total sample size, total number of events, and total number of discontinuations were included in the analysis. The results are summarized in Table 3.

**Table 3:** Multiple linear regression analysis.

Trial characteristics	Natural logarithm of FI			Natural logarithm of CFI		
	Regression coefficient	95% CI	*P* value	Regression coefficient	95% CI	*P* value
Biologics or small-molecule drugs	0.283	0.066, 0.501	0.011	−0.494	−0.857, −0.131	0.010
Primary or co-primary outcome	0.288	0.063, 0.513	0.013	0.459	0.067, 0.850	0.024
Total sample size	0.001	0.000, 0.002	0.021	0.001	0.000, 0.003	0.019
Total number of events	0.002	0.000, 0.005	0.055	–	–	–
Total number of discontinuations	0.001	0.000, 0.003	0.096	−0.002	−0.010, 0.007	0.690
Year of publication	0.019	−0.002, 0.041	0.077	−0.014	−0.099, 0.071	0.742
Impact factor	0.000	−0.001, 0.001	0.858	0.002	−0.001, 0.006	0.143
Phase 2	−0.151	−0.381, 0.079	0.194	0.049	−0.297, 0.394	0.774
Phase not mentioned	−0.007	−0.353, 0.338	0.966	−1.089	−1.782, −0.396	0.004
Modified intention-to-treat analysis	0.043	−0.123, 0.208	0.610	−0.057	−0.363, 0.248	0.701
Per-protocol analysis	0.157	−0.189, 0.503	0.370	−0.929	−1.550, −0.308	0.005

*R* ^2^: 0.670 for FI model, 0.809 for CFI model. The data represented by the dash indicates not applicable.

After adjustment for other study characteristics, the association between treatment type (biologics or small-molecule drugs vs FMT or SCT) and the natural logarithm of the FI remained statistically significant (*B *= 0.283, 95% CI: 0.066 to 0.501). Conversely, trials involving biologics or small-molecule drugs demonstrated greater instability compared with those involving FMT or SCT, with a negative regression coefficient of −0.494 (95% CI: −0.857 to −0.131) for the natural logarithm of the CFI. Primary or co-primary outcomes showed greater robustness compared with other outcomes in both the FI (*B *= 0.288, 95% CI: 0.063 to 0.513) and CFI (*B *= 0.459, 95% CI: 0.067 to 0.850) models. Additionally, the total sample size was significantly associated with both the natural logarithm of the FI (*B *= 0.001, 95% CI: 0.000 to 0.002) and the CFI (*B *= 0.001, 95% CI: 0.000 to 0.003). However, after control for other variables, the association between the total number of discontinuations and outcome robustness was no longer statistically significant.

## Discussion

IBD poses a great challenge to disease management in clinical practice owing to its complex pathophysiological characteristics. New therapeutic options provide new treatment options, particularly for patients who are not responding to conventional therapies. This study evaluated the robustness of existing RCTs investigating the efficacy of these novel therapies for IBD. By applying the FI and CFI, we assessed the robustness of binary and continuous outcomes, which, to some extent, compensated for the limitations of traditional statistical measures that rely merely on *P* values. Our findings also emphasized several critical factors influencing the robustness of these trials, including treatment type, significance of the primary outcome, and sample size. Unlike previous FI-related studies, this is the first study to have involved not only novel pharmacological therapies, but also emerging treatments, including FMT and SCT, in IBD. Furthermore, we employed multiple linear regression analysis to explore the independent associations between various study characteristics and both the FI and the CFI.

One of the main findings of our study was that RCTs evaluating biologics and small-molecule inhibitors demonstrated significantly higher robustness than did those evaluating FMT and SCT in terms of binary outcomes, which was independent of other influencing factors such as the sample size, the number of discontinuations, and the type of outcome. For continuous outcomes, the univariate analysis of the CFI showed a consistent trend toward greater robustness for pharmacologic therapies, although this difference did not reach statistical significance. However, this association was not maintained in the multiple regression analysis. We consider this discrepancy to reflect a methodological limitation, specifically that the multiple regression model that incorporated 11 variables but was fitted to only 36 studies was statistically underpowered and highly susceptible to overfitting. Therefore, under these constraints, the univariate comparison, despite not adjusting for confounders, offers a more stable and interpretable perspective on the observed pattern of results. This greater robustness observed for biologics and small-molecule inhibitors may be attributed to the more mature and standardized design of their clinical trials, in which outcome measures are well established and validated, leading to more reliable results [17]. Conversely, despite the promise of FMT and SCT, the evidence supporting their efficacy may be more susceptible to data fluctuations, possibly due to variability in the study protocols, including, but not limited to, patient selection criteria, delivery methods, donor type, and duration of treatment [18, 19]. Consequently, further research with more robust designs is essential to establish their clinical utility in IBD.

Furthermore, our results underscore the importance of sample size in ensuring study robustness. Larger trials generally exhibited more stable results, likely because of the increased statistical power, which reduced the influence of random variation. This is consistent with prior studies that emphasized the need for adequate sample sizes to obtain reliable results in clinical research [20–23]. Additionally, we observed that primary or co-primary outcomes exhibited greater stability than did secondary and other outcomes. As secondary and other outcomes were considered only when the primary outcomes were not statistically significant, researchers should interpret the efficacy of interventions based on these outcomes with caution, minimizing potential biases that could lead to false-positive results.

Regarding discontinuations in clinical trials, two previous studies on the FI in IBD trials yielded contrasting findings: one identified a positive correlation between the total number of discontinuations and the FI, while the other found no significant association [22, 23]. Our correlation analysis supported the former study by revealing a significant positive association between total discontinuation and FI, which might suggest that more discontinuations lead to more robust results. However, after accounting for confounding variables, including sample size, using multiple linear regression, total discontinuation was no longer an independent predictor of outcome robustness. This loss of significance suggests that the initial correlation was likely confounded. One possible explanation is that, in RCTs on novel IBD therapies, premature withdrawal is predominantly due to poor efficacy or disease progression, which minimizes the uncertainty typically associated with missing outcome data. Moreover, appropriate imputation methods are commonly applied to mitigate the impact of early discontinuation on the analysis. Nevertheless, we maintain that, when the number of discontinuations exceeds the FI, particularly when it is unrelated to efficacy, the resulting missing data could potentially alter the conclusions of the study.

However, some limitations of this study should be acknowledged. First, the exclusion of studies that lost statistical significance after *P*-value recalculation may have introduced selection bias, potentially overestimating the overall robustness in our analysis. These exclusions primarily resulted from differences in statistical tests between original studies and the standardized Fisher’s exact test used for FI calculation. A sensitivity analysis including these studies with an imputed FI of 0 confirmed the stability of the observed associations between fragility metrics and study characteristics. This consistency strengthens the primary findings while underscoring how statistical interpretations depend on the analytical method. This methodological sensitivity reinforces the value of FI as a supplementary measure that extends beyond sole reliance on *P* values. Second, calculation of the CFI relies on simulated datasets that assume a normal distribution, which may not align with the actual data distribution in the original trial, leading to a discrepancy between the estimated and true CFI values. To overcome this limitation in future research, we recommend that primary studies should compute and report the CFI directly from the original trial data alongside *P* values. This practice would permit a more reliable and meaningful aggregation of fragility estimates in subsequent evidence synthesis. Furthermore, the interpretation of our CFI analysis is constrained by the limited number of studies reporting eligible continuous outcomes. This scarcity reflects the current research landscape in which such outcomes remain less commonly adopted than are dichotomous endpoints. The resulting sample-size limitation affects the precision of the CFI estimates and may have reduced the statistical power to detect subtle associations between study characteristics and outcome robustness. Therefore, the results pertaining to the CFI should be interpreted with caution and are considered exploratory and preliminary. Their confirmation will require future studies with larger datasets. Third, owing to the limitations of the available data, our study did not calculate a specific cutoff value for the FI. Future studies should include a larger number of trials to establish a universally applicable FI cutoff value and define the robustness of trials.

## Conclusion

Therefore, the trials involving biologics and small-molecule inhibitors may be more robust than those involving FMT and SCT. Findings from trials with statistically insignificant primary outcomes should be interpreted with caution. Future RCTs evaluating novel therapies for IBD should add the FI to the *P* value when reporting results, enhancing efficacy assessments. Increasing the sample size and ensuring strict discontinuation management are critical for reliability.

## Supplementary Material

goag051_Supplementary_Data
