# Supplementary material for: Assessing the robustness of clinical trials regarding novel therapies in inflammatory bowel disease
Source: Gastroenterol Rep (Oxf). 2026 Jul 16;14:goag051. doi: 10.1093/gastro/goag051 (PMC13375628; doi:10.1093/gastro/goag051)
Supplement: goag051_Supplementary_Data [file goag051_supplementary_data.pdf]

Supplement materials

Supplementary tables 2

Supplementary Table 1. The search strategy 2

Supplementary Table 2. The studies that lost significance after recalculation 7

Supplementary Table 3. Characteristics of included studies for fragile index and fragility quotient 8

Supplementary Table 4. Characteristics of included studies for continuous fragile index and continuous fragility quotient 12

Supplementary Table 5. Data of included trials for fragile index and fragility quotient .. 14

Supplementary Table 6. Data of included trials for continuous fragile index and continuous fragility quotient 23

Supplementary Table 7. Associations between trial characteristics and fragility index (including trials with FI=0) 26

REFERENCES 29

删除[Jieqi Zheng]: 4

Supplementary tables

Supplementary Table 1. The search strategy

**PubMed**	
#1 Biologic	("infliximab"[Title/Abstract] OR "adalimumab"[Title/Abstract] OR "Certolizumab"[Title/Abstract] OR "ustekinumab"[Title/Abstract] OR "vedolizumab"[Title/Abstract] OR "golimumab"[Title/Abstract] OR "etrolizumab"[Title/Abstract] OR "ontamalimab"[Title/Abstract] OR "mirikizumab"[Title/Abstract] OR "risankizumab"[Title/Abstract] OR "ontamalimab"[Title/Abstract] OR "guselkumab"[Title/Abstract] OR ("antib technol j"[Journal] OR "anti"[All Fields] OR ("antib technol j"[Journal] OR "anti"[All Fields]) OR ("antagonist"[All Fields] OR "antagonists and inhibitors"[MeSH Subheading] OR ("antagonists"[All Fields] AND "inhibitors"[All Fields]) OR "antagonists and inhibitors"[All Fields] OR "antagonists"[All Fields]) OR ("antagonists and inhibitors"[MeSH Subheading] OR ("antagonists"[All Fields] AND "inhibitors"[All Fields]) OR "antagonists and inhibitors"[All Fields] OR "inhibitors"[All Fields] OR "inhibitor"[All Fields] OR "inhibitor s"[All Fields]))) AND ("TNF"[All Fields] OR ("tumor necrosis factor alpha"[MeSH Terms] OR ("tumor"[All Fields] AND "necrosis"[All Fields] AND "factor alpha"[All Fields]) OR "tumor necrosis factor alpha"[All Fields] OR ("TNF"[All Fields] AND "alpha"[All Fields]) OR "tnf alpha"[All Fields]) OR ("tumor necrosis factor alpha"[MeSH Terms] OR ("tumor"[All Fields] AND "necrosis"[All Fields] AND "factor alpha"[All Fields]) OR "tumor necrosis factor alpha"[All Fields] OR "tnfalpha"[All Fields]) OR ("tumor necrosis factor alpha"[MeSH Terms] OR ("tumor"[All Fields] AND "necrosis"[All Fields] AND "factor alpha"[All Fields]) OR "tumor necrosis factor alpha"[All Fields] OR ("TNF"[All Fields] AND "alpha"[All Fields]) OR "tnf alpha"[All Fields]) OR ("tumour necrosis factor"[All Fields] OR "tumor necrosis factor alpha"[MeSH Terms] OR ("tumor"[All Fields] AND "necrosis"[All Fields] AND "factor alpha"[All Fields]) OR "tumor necrosis factor alpha"[All Fields] OR ("tumor"[All Fields] AND "necrosis"[All Fields] AND "factor"[All Fields]) OR "tumor necrosis factor"[All Fields]) OR ("tumour necrosis factor"[All Fields] OR "tumor necrosis factor alpha"[MeSH Terms] OR ("tumor"[All Fields] AND "necrosis"[All Fields] AND "factor alpha"[All Fields]) OR "tumor necrosis factor alpha"[All Fields] OR ("tumor"[All Fields] AND "necrosis"[All Fields] AND "factor"[All Fields]) OR "tumor necrosis factor"[All Fields]) OR "IL"[All Fields] OR ("interleukine"[All Fields] OR "interleukines"[All Fields] OR "interleukins"[MeSH Terms] OR "interleukins"[All Fields] OR "interleukin"[All Fields]) OR ("integrin s"[All Fields] OR "integrins"[MeSH Terms] OR "integrins"[All Fields] OR "integrin"[All Fields]))
#2 Small molecule drug	((((((((((("Sphingosine 1 Phosphate Receptor Modulators"[Mesh]) OR (ozanimod[Title/Abstract])) OR (etrasimod[Title/Abstract])) OR (Fingolimod[Title/Abstract])) OR (Amiselimod[Title/Abstract])) OR

	(Obefazimod[Title/Abstract])) OR (((((SMAD7[Title/Abstract]) AND (((((anti-) OR (anti)) OR (antagonist)) OR (inhibitor)))) OR (Mongersen[Title/Abstract])) OR (GED-0301[Title/Abstract])) OR (Celgene[Title/Abstract]))) OR ((((PDE4[Title/Abstract]) AND (((((anti-) OR (anti)) OR (antagonist)) OR (inhibitor)))) OR ("Phosphodiesterase 4 Inhibitors"[Mesh])) OR (apremilast[Title/Abstract]))) OR (((("Integrins"[Mesh]) AND (((((anti-) OR (anti)) OR (antagonist)) OR (inhibitor)))) OR (AJM-300[Title/Abstract])) OR (Carotegrast[Title/Abstract]))) OR (((("Intercellular Adhesion Molecule-1"[Mesh]) OR (ICAM[Title/Abstract])) AND (((((anti-) OR (anti)) OR (antagonist)) OR (inhibitor)))) OR (Alicaforsen[Title/Abstract]))) OR ((("Toll-Like Receptors"[Mesh]) AND (((((anti-) OR (anti)) OR (antagonist)) OR (inhibitor)))) OR (Cobitolimod[Title/Abstract]))) OR ((((((((((((((JAK[Title/Abstract]) OR (Janus Kinase[Title/Abstract])) AND (((((anti-) OR (anti)) OR (antagonist)) OR (inhibitor)))) OR ("Janus Kinase Inhibitors"[Mesh])) OR (tofacitinib[Title/Abstract])) OR (filgotinib[Title/Abstract])) OR (upadacitinib[Title/Abstract])) OR (TD-1473[Title/Abstract])) OR (Pf-06651600[Title/Abstract])) OR (Pf-06700841[Title/Abstract])) OR (Brepocitinib[Title/Abstract])) OR (Ruxolitinib[Title/Abstract])) OR (Baricitinib[Title/Abstract])) OR (Peficitinib[Title/Abstract]))
#3 Fecal microbiota transplantation	(((((((((((((("Fecal Microbiota Transplantation"[Mesh]) OR (Fecal Microbiota Transplant[Title/Abstract])) OR (Microbiota Transplant, Fecal[Title/Abstract])) OR (Fecal Microbiome Transplantation[Title/Abstract])) OR (Fecal Transplant[Title/Abstract])) OR (Donor Feces Infusion[Title/Abstract])) OR (Intestinal Microbiome Transplant[Title/Abstract])) OR (Intestinal Microbiota Transfer[Title/Abstract])) OR (Fecal Microbiota Transfer[Title/Abstract])) OR (bacteriotherap*[Title/Abstract])) OR (colonic restoration[Title/Abstract])) OR (flora reconstitution[Title/Abstract])) OR (RBX2660[Title/Abstract])) OR (FMT[Title/Abstract])) OR ((((((((((Fecal[Title/Abstract]) OR (Faecal[Title/Abstract])) OR (microbiota[Title/Abstract])) OR (microflora[Title/Abstract])) OR (feces[Title/Abstract])) OR (faeces[Title/Abstract])) OR (stool[Title/Abstract])) OR (bacteria[Title/Abstract])) OR (bacterio*[Title/Abstract])) AND (((((((((((transplant*[Title/Abstract]) OR (transfus*[Title/Abstract])) OR (implant*[Title/Abstract])) OR (instillation[Title/Abstract])) OR (donor*[Title/Abstract])) OR (enema[Title/Abstract])) OR (reconstitution[Title/Abstract])) OR (infusion*[Title/Abstract])) OR (therap*[Title/Abstract])) OR (transfer*[Title/Abstract])) OR (treat*[Title/Abstract])))
#4 Stem cell	(((((((((((((((((((((((((((((((((((((((Mesenchymal Stem Cell[Title/Abstract])) OR (Bone Marrow Mesenchymal Stem Cells[Title/Abstract])) OR (Bone Marrow Mesenchymal Stem Cell[Title/Abstract])) OR (Bone Marrow Stromal Cells[Title/Abstract])) OR (Bone Marrow Stromal Cell[Title/Abstract])) OR (Multipotent Bone Marrow Stromal Cell[Title/Abstract])) OR (Multipotent Bone Marrow Stromal Cells[Title/Abstract])) OR (Adipose-Derived Mesenchymal Stem

	Cells[Title/Abstract])) OR (Adipose Derived Mesenchymal Stem Cells[Title/Abstract])) OR (Adipose-Derived Mesenchymal Stromal Cells[Title/Abstract])) OR (Adipose Derived Mesenchymal Stromal Cells[Title/Abstract])) OR (Adipose-Derived Mesenchymal Stem Cell[Title/Abstract])) OR (Adipose Derived Mesenchymal Stem Cell[Title/Abstract])) OR (Adipose Tissue-Derived Mesenchymal Stem Cell[Title/Abstract])) OR (Adipose Tissue Derived Mesenchymal Stem Cell[Title/Abstract])) OR (Adipose Tissue-Derived Mesenchymal Stem Cells[Title/Abstract])) OR (Adipose Tissue Derived Mesenchymal Stem Cells[Title/Abstract])) OR (Adipose Tissue-Derived Mesenchymal Stromal Cells[Title/Abstract])) OR (Adipose Tissue Derived Mesenchymal Stromal Cells[Title/Abstract])) OR (Adipose Tissue-Derived Mesenchymal Stromal Cell[Title/Abstract])) OR (Adipose Tissue Derived Mesenchymal Stromal Cell[Title/Abstract])) OR (Mesenchymal Stromal Cells[Title/Abstract])) OR (Mesenchymal Stromal Cell[Title/Abstract])) OR (Multipotent Mesenchymal Stromal Cells[Title/Abstract])) OR (Multipotent Mesenchymal Stromal Cell[Title/Abstract])) OR (Mesenchymal Progenitor Cell[Title/Abstract])) OR (Mesenchymal Progenitor Cells[Title/Abstract])) OR (Wharton Jelly Cells[Title/Abstract])) OR (Wharton's Jelly Cells[Title/Abstract])) OR (Wharton's Jelly Cell[Title/Abstract])) OR (Whartons Jelly Cells[Title/Abstract])) OR (Bone Marrow Stromal Stem Cells[Title/Abstract])) OR ("Stem Cells"[Mesh])) OR ("Stem Cell Transplantation"[Mesh])) OR (SCT[Title/Abstract])) OR ("Hematopoietic Stem Cells"[Mesh])) OR ("Bone Marrow Transplantation"[Mesh])) OR (BMT[Title/Abstract])) OR ((((hematopoietic[Title/Abstract]) OR (haematopoietic[Title/Abstract])) OR (bone marrow[Title/Abstract])) AND (((((autologous[Title/Abstract]) OR (autotransfusion[Title/Abstract])) OR (auto-transfus*[Title/Abstract])) OR (autograF*[Title/Abstract])) OR (allogenic[Title/Abstract])))
#5	Or/#1-4
#6 Randomized controlled trial	((((((random*[Title/Abstract]) OR (random?ed[Title/Abstract])) OR (clinical trial[Title/Abstract])) OR (controlled trial[Title/Abstract])) OR (Randomized Controlled Trial[Publication Type])) OR (Clinical Trial[Publication Type])) NOT (animal[Filter])
#7 Inflammatory bowel diseases	((((((((((((("Inflammatory Bowel Diseases"[Mesh]) OR (Inflammatory Bowel Disease[Title/Abstract])) OR ("Crohn Disease"[Mesh])OR (Crohn's Enteritis[Title/Abstract])) OR (Regional Enteritis[Title/Abstract])) OR (Crohn's Disease[Title/Abstract])) OR (Crohns Disease[Title/Abstract])) OR (Granulomatous Enteritis[Title/Abstract])) OR (Regional Enteritis[Title/Abstract])) OR (Ileocolitis[Title/Abstract])) OR (Granulomatous Colitis[Title/Abstract])) OR (Terminal Ileitis[Title/Abstract])) OR (Regional Ileitis[Title/Abstract])) OR ("Colitis, Ulcerative"[Mesh])) OR (Ulcerative Colitis[Title/Abstract])) OR (Colitis Gravis[Title/Abstract])
#8 Publication	"2014/1/1"[Date - Publication] : "2024/11/10"[Date - Publication]

date	
#9 Excluded Publication type	(((((((((Case Reports[Publication Type]) OR (Comment[Publication Type])) OR (Editorial[Publication Type])) OR (English Abstract[Publication Type])) OR (Letter[Publication Type])) OR (Meta-Analysis[Publication Type])) OR (News[Publication Type])) OR (Observational study[Publication Type])) OR (Guideline[Publication Type])) OR (Review[Publication Type])
#10	And/#5-8
#11	#10 NOT #9
**Web of Science**	
#1 Biologic	(TS=(infliximab OR adalimumab OR Certolizumab OR ustekinumab OR vedolizumab OR golimumab OR etrolizumab OR ontamalimab OR mirikizumab OR risankizumab OR ontamalimab OR guselkumab)) OR (TS=(anti- OR anti OR antagonist OR inhibitor)) AND TS=(TNF OR TNF-alpha OR TNF-α OR tumour necrosis factor OR tumor necrosis factor OR IL OR interleukin OR integrin)
#2 Small molecule drug	(TS=("Sphingosine 1 Phosphate Receptor Modulators" OR ozanimod OR etrasimod OR Fingolimod OR Amiselimod OR Obefazimod)) OR ((((TS=(PDE4)) AND TS=(anti- OR anti OR antagonist OR inhibitor)) OR TS=("Phosphodiesterase 4 Inhibitors")) OR TS=(apremilast)) OR (((TS=("Integrins")) AND TS=(anti- OR anti OR antagonist OR inhibitor)) OR TS=(AJM-300 OR Carotegrast)) OR (((((TS=(SMAD7)) AND TS=(anti- OR anti OR antagonist OR inhibitor)) OR TS=(Mongersen)) OR TS=(GED-0301)) OR TS=(Celgene)) OR (((TS=("Intercellular Adhesion Molecule-1" OR ICAM)) AND TS=(anti- OR anti OR antagonist OR inhibitor)) OR TS=(Alicaforsen)) OR (((TS=("Toll-Like Receptors")) AND TS=(anti- OR anti OR antagonist OR inhibitor)) OR TS=(Cobitolimod)) OR (((TS=(JAK OR Janus Kinase)) AND TS=(anti- OR anti OR antagonist OR inhibitor)) OR TS=("Janus Kinase Inhibitors" OR tofacitinib OR filgotinib OR upadacitinib OR TD-1473 OR Pf-06651600 OR Pf-06700841 OR Brepocitinib OR Ruxolitinib OR Baricitinib OR Peficitinib))
#3 Fecal microbiota transplantation	((TS=(Fecal OR Faecal OR microbiota OR microflora OR feces OR faeces OR stool OR bacteria OR bacterio*)) AND TS=(transplant* OR transfus* OR implant* OR instillation OR donor* OR enema OR reconstitution OR infusion* OR therap* OR transfer* OR treat*)) OR TS=("Fecal Microbiota Transplantation" OR Fecal Microbiota Transplant OR Microbiota Transplant, Fecal OR Fecal Microbiome Transplantation OR Fecal Transplant OR Donor Feces Infusion OR Intestinal Microbiome Transplant OR Intestinal Microbiota Transfer OR Fecal Microbiota Transfer OR bacteriotherap* OR colonic restoration OR flora reconstitution OR RBX2660 OR FMT)
#4 Stem cell	(TS=(Mesenchymal Stem Cell OR Bone Marrow Mesenchymal Stem Cells OR Bone Marrow Mesenchymal Stem Cell OR Bone Marrow Stromal Cells OR Bone Marrow Stromal Cell OR Multipotent Bone Marrow Stromal Cell OR Multipotent Bone Marrow Stromal Cells OR Adipose-Derived Mesenchymal Stem Cells OR Adipose Derived Mesenchymal Stem Cells OR Adipose-Derived Mesenchymal

	Stromal Cells OR Adipose Derived Mesenchymal Stromal Cells OR Adipose-Derived Mesenchymal Stem Cell OR Adipose Derived Mesenchymal Stem Cell OR Adipose Tissue-Derived Mesenchymal Stem Cell OR Adipose Tissue Derived Mesenchymal Stem Cell OR Adipose Tissue-Derived Mesenchymal Stem Cells OR Adipose Tissue Derived Mesenchymal Stem Cells OR Adipose Tissue-Derived Mesenchymal Stromal Cells OR Adipose Tissue Derived Mesenchymal Stromal Cells OR Adipose Tissue-Derived Mesenchymal Stromal Cell OR Adipose Tissue Derived Mesenchymal Stromal Cell OR Mesenchymal Stromal Cells OR Mesenchymal Stromal Cell OR Multipotent Mesenchymal Stromal Cells OR Multipotent Mesenchymal Stromal Cell OR Mesenchymal Progenitor Cell OR Mesenchymal Progenitor Cells OR Wharton Jelly Cells OR Wharton's Jelly Cells OR Wharton's Jelly Cell OR Whartons Jelly Cells OR Bone Marrow Stromal Stem Cells OR "Stem Cells" OR "Stem Cell Transplantation" OR SCT OR "Hematopoietic Stem Cells" OR "Bone Marrow Transplantation" OR BMT OR hematopoietic OR haematopoietic OR autotransfusion OR auto-transfus* OR autograF* OR allogenic)) OR TS=((bone marrow) AND (autologous))
#5	Or/#1-4
#6 Randomized controlled trial	TS=(random* OR random?ed OR clinical trial OR controlled trial OR Randomized Controlled Trial OR Clinical Trial)
#7 Inflammatory bowel diseases	TS=("Inflammatory Bowel Diseases" OR Inflammatory Bowel Disease OR "Crohn Disease"OR Crohn's Enteritis OR Regional Enteritis OR Crohn's Disease OR Crohns Disease OR Granulomatous Enteritis OR Regional Enteritis OR Ileocolitis OR Granulomatous Colitis OR Terminal Ileitis OR Regional Ileitis OR "Colitis, Ulcerative" OR Ulcerative Colitis OR Colitis Gravis
#8 Publication date	DOP=(2014-01-01/2024-11-10)
#9 Excluded Publication type	Review Article or Abstract or Meeting or Awarded Grant or Editorial Material or Case Report or Dissertation Thesis or Book or Letter or Early Access or Reference Material or Correction or Retracted Publication or News (Exclude – Document Types)
#10	And/#5-9
**The last search time: 2024/11/10**	

Supplementary Table 2. The studies that lost significance after recalculation

Title	Author	Year	Sample size	Outcome	Reported p-value or 95% CI
To Determine the Frequency of Clinical Remission Induction with Versus Without Fecal Microbiota Transplant in Treatment of Active Ulcerative Colitis	A. Abou Bakr[1]	2024	90	Patients with a Mayo score of ≤2	P = 0.003
Fecal Microbial Transplantation versus Mesalamine Enema for Treatment of Active Left-Sided Ulcerative Colitis-Results of a Randomized Controlled Trial	J.Březina[2]	2021	43	Clinical remission (Mayo score ≤ 2, with no subscore > 1)	95% CI: (−7.6%, 48.9%)
Long-term efficacy and safety of monotherapy with a single fresh fecal microbiota transplant for recurrent active ulcerative colitis: a prospective randomized pilot study	H.Fang[3]	2021	20	Steroid free remission (total Mayo score of 2 with an endoscopic Mayo score of 1 or less)	Not mentioned
Efficacy and safety of abrilumab, an $\alpha 4 \beta 7$ integrin inhibitor, in Japanese patients with moderate-to-severe ulcerative colitis: a phase II study	T.Hibi[4]	2019	45	Clinical remission at week 8 (total Mayo score ≤ 2 points with no individual subscore >1 point)	Not mentioned
Cobitolimod for moderate-to-severe, left-sided ulcerative colitis (CONDUCT): a phase 2b randomised, double-blind, placebo-controlled, dose-ranging induction trial	R.Atreya[5]	2020	86	Clinical remission (Mayo subscores for rectal bleeding of 0, for stool frequency of 0 or 1 [with ≥1-point decrease from baseline], and for endoscopy of 0 or 1)	P = 0.025
Ozanimod Induction and Maintenance Treatment for Ulcerative Colitis	W.J.Sandborn[6]	2016	132	Clinical remission (Mayo Clinic score ≤2, with no subscore >1)	P = 0.048

CI, confidence interval.

Supplementary Table 3. Characteristics of included studies for fragile index and fragility quotient

ID	Author	Year	Disease	Type of therapy	Center	Trial phase	Blinding type	Treatment	Control	Type of outcome
1	S. Danese[7]	2019	Moderately to severely active CD	Biologics	Multicenter	2	Double-blind	PF-04236921	Placebo	Primary
2	P. Rutgeerts[8]	2018	Moderately to severely active CD	Biologics	Multicenter	3	Double-blind	Ustekinumab	Placebo	Secondary
3	S. Vermeire[9]	2017	Active UC	Biologics	Multicenter	2	Double-blind	PF-00547659	Placebo	Primary
4	S. Vermeire[10]	2014	Moderately to severely active UC	Biologics	Multicenter	2	Double-blind	Etrolizumab	Placebo	Primary
5	B. G. Feagan[11]	2017	Moderately to severely active CD	Biologics	Multicenter	2	Double-blind	Risankizumab	Placebo	Primary
6	G. D'Haens[12]	2022	Moderately to severely active CD	Biologics	Multicenter	3	Double-blind	Risankizumab	Placebo	Coprimary
7	M. Ferrante[13]	2022	Moderately to severely active CD	Biologics	Multicenter	3	Double-blind	Risankizumab	Placebo	Coprimary
8	B. G. Feagan[14]	2016	Moderately to severely active CD	Biologics	Multicenter	3	Double-blind	Ustekinumab	Placebo	Primary
9	B. E. Sands[15]	2019	Moderately to severely UC	Biologics	Multicenter	3	Double-blind	Ustekinumab	Placebo	Primary
10	B. E. Sands[16]	2014	Moderately to severely CD	Biologics	Multicenter	3	Double-blind	Vedolizumab	Placebo	Secondary
11	B. E. Sands[17]	2017	Moderately to severely CD	Biologics	Multicenter	2	Double-blind	MEDI2070	Placebo	Primary
12	W. J. Sandborn[18]	2020	Moderately to severely active UC	Biologics	Multicenter	2	Double-blind	Mirikizumab	Placebo	Primary

13	L. Peyrin - Biroulet[19]	2023	Moderately to severely active UC	Biologics	Multicenter	2	Double-blind	Guselkumab	Placebo	Primary
14	W. J. Sandborn[20]	2014	Moderately to severely UC	Biologics	Multicenter	3	Double-blind	Golimumab	Placebo	Primary
15	J. F. Colombel[21]	2014	Moderately to severely ileocolonic CD	Biologics	Multicenter	3	Double-blind	Adalimumab	Placebo	Coprimary
16	S. Vermeire[22]	2023	Moderately to severely UC	Biologics	Multicenter	3	Double-blind	Ontamalimab	Placebo	Primary
17	M. Allez[23]	2023	Moderately to severely active CD	Biologics	Multicenter	2	Double-blind	Tesnatilimab	Placebo	Others
18	Y. Suzuki[24]	2014	Moderately to severely active UC	Biologics	Multicenter	3	Double-blind	Adalimumab	Placebo	Primary
19	T. Hibi[25]	2017	Moderately to severely active UC	Biologics	Multicenter	3	Double-blind	Golimumab	Placebo	Primary
20	S. Motoya[26]	2019	Moderately to severely UC	Biologics	Multicenter	3	Double-blind	vedolizumab	Placebo	Primary
21	X.-L. Jiang[27]	2015	Moderately to severely active UC	Biologics	Single-center	Not mentioned	Double-blind	Infliximab	Placebo	Primary
22	S. Vermeir[28]	2023	Moderately to severely active UC	Biologics	Multicenter	3	Double-blind	Upadacitinib	Placebo	Primary
23	B. E. Sands[29]	2024	Moderately to severely active UC	Biologics	Multicenter	2	Double-blind	Tulisokibart	Placebo	Primary
24	E. Louis[30]	2024	UC	Biologics	Multicenter	3	Double-blind	Risankizumab	Placebo	Primary
25	E. V. Loftus[31]	2023	Moderately to severely active CD	Small molecule drug	Multicenter	3	Double-blind	Upadacitinib	Placebo	Primary
26	W. J. Sandborn[32]	2021	Moderately to severely active UC	Small molecule drug	Multicenter	3	Double-blind	Ozanimod	Placebo	Primary

27	B. Chen[33]	2022	Moderately to severely active UC	Small molecule drug	Multicenter	2	Double-blind	Ivarmacitinib	Placebo	Secondary
28	S. Danese[34]	2022	Moderately to severely active UC	Small molecule drug	Multicenter	3	Double-blind	Upadacitinib	Placebo	Primary
29	W. J. Sandborn[35]	2017	Moderately to severely active UC	Small molecule drug	Multicenter	3	Double-blind	Tofacitinib	Placebo	Primary
30	G. Monteleone[36]	2015	Moderately to severely CD	Small molecule drug	Multicenter	2	Double-blind	Mongersen (GED-0301)	Placebo	Primary
31	K. Matsuoka[37]	2022	Moderately active UC	Small molecule drug	Multicenter	3	Double-blind	AJM300	Placebo	Primary
32	W. J. Sandborn[38]	2020	Moderately to severely CD	Small molecule drug	Multicenter	2	Double-blind	Upadacitinib	Placebo	Primary
33	W. J. Sandborn[39]	2020	Moderately to severely active UC	Small molecule drug	Multicenter	2	Double-blind	Upadacitinib	Placebo	Primary
34	N. Yoshimura[40]	2015	Moderately active UC	Small molecule drug	Multicenter	2	Double-blind	AJM300	Placebo	Primary
35	S. Danese[41]	2020	Active UC	Small molecule drug	Multicenter	2	Double-blind	Apremilast	Placebo	Primary
36	B. E. Sands[42]	2024	Moderately to severely UC	Small molecule drug	Multicenter	3	Double-blind	Ozanimod	Placebo	Primary
37	A. Singh[43]	2024	Acute severe UC	Small molecule drug	Single-center	Not mentioned	Double-blind	Tofacitinib	Placebo	Primary
38	J. Hu[44]	2016	Moderately to severely UC	Stem cell	Single-center	1/2	Double-blind	MSC infusion	Saline solution	Primary
39	G. Y. Melmed[45]	2015	Moderately to severely CD	Stem cell	Multicenter	1/2	Double-blind	Human placenta-derived cells (PDA-001)	Vehicle control without cells	Primary

40	J. Panés[46]	2018	Complex perianal fistulas in CD	Stem cell	Multicenter	3	Double-blind	Cx601 cells	Saline solution	Secondary
41	J. Panes[47]	2016	Complex perianal fistulas in CD	Stem cell	Multicenter	3	Double-blind	Cx601 cells	Saline solution	Primary
42	S. Kedia[48]	2022	Mild to moderate UC	FMT	Single-center	Not mentioned	Open-labelled	Faecal microbiota transplantation with anti-inflammatory diet (FMT-AID)	Optimized standard medical therapy (SMT)	Primary
43	A. Sood[49]	2019	UC	FMT	Single-center	2/3	Double-blind	Colonoscopic infusion of FMT	Placebo	Secondary
44	P. Moayyedi[50]	2015	Active UC	FMT	Multicenter	2	Double-blind	FMT enema	Placebo (water enema)	Primary
45	C. Haifer[51]	2022	Active UC	FMT	Multicenter	2	Double-blind	Lyophilised FMT	Placebo	Primary
46	S. Paramsothy[52]	2017	Active UC	FMT	Multicenter	2	Double-blind	FMT enema	Placebo (water enema)	Primary
47	S. P. Costello[53]	2019	Mild to moderate UC	FMT	Multicenter	2	Double-blind	Anaerobically prepared pooled donor FMT	Autologous FMT	Primary

ID, exclusive code assigned to each study; CD, Crohn's disease; UC, ulcerative colitis; FMT, fecal microbiota transplantation.

Supplementary Table 4. Characteristics of included studies for continuous fragile index and continuous fragility quotient

ID	Author	Year	Disease	Type of therapy	Center	Trial phase	Blinding type	Treatment	Control	Type of outcome
1	W. J. Sandborn[54]	2022	Moderately to severely active CD	Biologics	Multicenter	2	Double-blind	Guselkumab	Placebo	Primary
2	P. Rutgeerts[8]	2018	Moderately to severely active CD	Biologics	Multicenter	3	Double-blind	Ustekinumab	Placebo	Primary
3	S. Vermeire[9]	2017	Active UC	Biologics	Multicenter	2	Double-blind	PF-00547659	Placebo	Secondary
4	B. E. Sands[15]	2019	Moderately to severely UC	Biologics	Multicenter	3	Double-blind	Ustekinumab	Placebo	Others
5	T. Hibi[25]	2017	Moderately to severely active UC	Biologics	Multicenter	3	Double-blind	Golimumab	Placebo	Others
6	S. Vermeire[55]	2022	Moderately to severely active UC	Small molecule drug	Multicenter	2	Double-blind	ABX464	Placebo	Primary
7	W. J. Sandborn[56]	2020	UC	Small molecule drug	Multicenter	2	Double-blind	Etrasimod	Placebo	Primary
8	B. Chen[33]	2022	Moderately to severely active UC	Small molecule drug	Multicenter	2	Double-blind	Ivarmacitinib	Placebo	Secondary

9	G. Monteleone[36]	2015	Moderately to severely CD	Small molecule drug	Multicenter	2	Double-blind	Mongersen (GED-0301)	Placebo	Secondary
10	W. J. Sandborn[38]	2020	Moderately to severely CD	Small molecule drug	Multicenter	2	Double-blind	Upadacitinib	Placebo	Secondary
11	W. J. Sandborn[39]	2020	Moderately to severely active UC	Small molecule drug	Multicenter	2	Double-blind	Upadacitinib	Placebo	Secondary
12	N. Yoshimura[40]	2015	Moderately active UC	Small molecule drug	Multicenter	2	Double-blind	AJM300	Placebo	Others
13	J. Zhang[57]	2018	CD	Stem cell	Single-center	1/2	Open-labelled	Umbilical cord mesenchymal stem cell	Placebo	Not mentioned
14	C. Zhou[58]	2020	Crohn's fistula-in-ano	Stem cell	Single-center	Not mentioned	Open-labelled	Autologous adipose derived stem cell	Incision-thread-drawing procedure	Secondary
15	C. J. Hawkey[59]	2015	Refractory CD	Stem cell	Multicenter	3	Open-labelled	Hematopoietic stem cell transplantation	Standard treatment	Secondary
16	A. Sood[49]	2019	UC	FMT	Single-center	2/3	Double-blind	Colonoscopic infusion of FMT	Placebo	Others

ID, exclusive code assigned to each study; CD, Crohn's disease; UC, ulcerative colitis; FMT, fecal microbiota transplantation.

Supplementary Table 5. Data of included trials for fragile index and fragility quotient

ID	Outcome	Follow up time	Sample size			Number of events			Discontinuation			FI	FQ
			Total	Interventional	Control	Total	Interventional	Control	Total	Interventional	Control		
1	CDAI-70 response	8 weeks	111	54	57	42	26	16	24	13	11	1	0.009
2	Endoscopic response	44 weeks	53	29	24	8	1	7	null	null	null	1	0.019
3.1	Clinical and endoscopic remission	12 weeks	145	72	73	14	12	2	6	1	5	3	0.021
3.2	Clinical and endoscopic remission	12 weeks	144	71	73	13	11	2	8	3	5	2	0.014
4	Clinical remission	10 weeks	80	39	41	8	8	0	6	4	2	3	0.038
5	Clinical remission	12 weeks	80	41	39	21	15	6	7	1	6	1	0.013
6.1	CDAI clinical remission	12 weeks	378	191	187	117	80	37	32	6	26	23	0.061
6.2	CDAI clinical remission	12 weeks	378	191	187	114	77	37	33	7	26	20	0.053

6.3	Stool frequency and abdominal pain score clinical remission	12 weeks	378	191	187	102	66	36	32	6	26	11	0.029
6.4	Stool frequency and abdominal pain score clinical remission	12 weeks	378	191	187	112	76	36	33	7	26	20	0.053
6.5	Endoscopic response	12 weeks	378	191	187	76	55	21	32	6	26	17	0.045
6.6	Endoscopic response	12 weeks	378	191	187	86	65	21	33	7	26	25	0.066
7.1	CDAI clinical remission	52 weeks	321	157	164	154	87	67	33	13	20	6	0.019
7.2	Endoscopic response	52 weeks	321	157	164	110	74	36	33	13	20	23	0.072
7.3	Endoscopic response	52 weeks	305	141	164	102	66	36	37	17	20	23	0.075
8.1	Clinical	6 weeks	492	245	247	137	84	53	19	9	10	12	0.024

	response											
8.2	Clinical response	6 weeks	496	249	247	137	84	53	24	14	10	11	0.022
8.3	Clinical response	6 weeks	418	209	209	168	108	60	21	9	12	28	0.067
8.4	Clinical response	6 weeks	418	209	209	176	116	60	14	2	12	35	0.084
8.5	Clinical remission	44 weeks	260	129	131	110	63	47	22	9	13	1	0.004
8.6	Clinical remission	44 weeks	259	128	131	115	68	47	27	14	13	7	0.027
9.1	Clinical remission	8 weeks	639	320	319	67	50	17	44	16	28	16	0.025
9.2	Clinical remission	8 weeks	641	322	319	67	50	17	40	12	28	16	0.025
9.3	Clinical remission	44 weeks	347	172	175	108	66	42	21	11	10	7	0.020
9.4	Clinical remission	44 weeks	351	176	175	119	77	42	18	8	10	17	0.048
10.1	Clinical remission	10 weeks	315	158	157	61	42	19	19	7	12	8	0.025
10.2	CDAI-100 response	6 weeks	315	158	157	97	62	35	19	7	12	10	0.032
10.3	Clinical remission	10 weeks	416	209	207	87	60	27	28	13	15	15	0.036
10.4	Clinical	10 weeks	416	209	207	49	32	17	28	13	15	2	0.005

	remission												
11	Clinical response	8 weeks	119	59	60	45	29	16	9	5	4	3	0.025
12	Clinical remission	12 weeks	125	62	63	17	14	3	5	2	3	3	0.024
13.1	Clinical response	12 weeks	206	101	105	91	62	29	6	2	4	21	0.102
13.2	Clinical response	12 weeks	212	107	105	94	65	29	5	1	4	20	0.094
14.1	Clinical response	54 weeks	305	151	154	119	71	48	72	31	41	8	0.026
14.2	Clinical response	54 weeks	305	151	154	123	75	48	79	38	41	11	0.036
15	Deep remission	52 weeks	123	62	61	12	12	0	15	10	5	4	0.033
16.1	Clinical remission	52 weeks	144	71	73	44	38	6	14	6	8	21	0.146
16.2	Clinical remission	52 weeks	168	82	86	44	33	11	9	1	8	12	0.071
17	Endoscopic response	12 weeks	117	62	55	28	20	8	7	3	4	1	0.009
18.1	Clinical response	8 weeks	186	90	96	85	50	35	8	4	4	4	0.022
18.2	Mucosal healing	8 weeks	186	90	96	74	44	30	8	4	4	3	0.016
19	Clinical	54 weeks	63	32	31	24	18	6	17	5	12	4	0.063

	response											
20	Clinical remission	60 weeks	83	41	42	36	23	13	35	11	24	2	0.024
21.1	Clinical response	8 weeks	82	41	41	45	30	15	28	8	20	6	0.073
21.2	Clinical response	8 weeks	82	41	41	47	32	15	28	8	20	8	0.098
22.1	Clinical remission	52 weeks	470	225	223	247	91	24	218	70	148	47	0.209
22.2	Clinical remission	52 weeks	470	233	223	247	125	24	197	49	148	76	0.326
23	Clinical remission	12 weeks	135	68	67	27	26	1	7	0	7	14	0.104
24.1	Clinical remission	52 weeks	362	179	183	118	72	46	30	12	18	10	0.028
24.2	Clinical remission	52 weeks	369	186	183	116	70	46	39	21	18	6	0.016
25.1	Clinical remission	12 weeks	334	169	165	88	63	25	41	23	18	20	0.060
25.2	Clinical remission	12 weeks	333	168	165	105	80	25	38	20	18	36	0.108
25.3	Endoscopic response	12 weeks	334	169	165	61	49	12	41	23	18	21	0.063
25.4	Endoscopic response	12 weeks	333	168	165	79	67	12	38	20	18	37	0.111

26	Clinical remission	52 weeks	457	230	227	127	85	42	149	46	103	23	0.050
27.1	Clinical remission	8 weeks	82	41	41	11	9	2	11	5	6	1	0.012
27.2	Clinical remission	8 weeks	82	41	41	12	10	2	8	2	6	1	0.012
27.3	Clinical remission	8 weeks	82	41	41	12	10	2	11	5	6	1	0.012
27.4	Endoscopic improvement	8 weeks	82	41	41	21	15	6	11	5	6	1	0.012
28.1	Clinical remission	52 weeks	297	148	149	81	63	18	147	49	98	29	0.098
28.2	Clinical remission	52 weeks	303	154	149	98	80	18	131	33	98	42	0.139
29.1	Remission	52 weeks	396	198	198	90	68	22	232	87	145	28	0.071
29.2	Remission	52 weeks	395	197	198	102	80	22	215	70	145	40	0.101
30.1	Clinical remission	2 weeks	82	40	42	32	22	10	2	1	1	4	0.049
30.2	Clinical remission	2 weeks	85	43	42	38	28	10	2	1	1	8	0.094
31	Clinical response	8 weeks	203	102	101	67	46	21	27	11	16	11	0.054
32.1	Endoscopic remission	16 weeks	73	36	37	8	8	0	17	7	10	3	0.041

32.2	Endoscopic remission	16 weeks	72	35	37	5	5	0	15	5	10	1	0.014
33.1	Clinical remission	8 weeks	95	49	46	7	7	0	9	4	5	1	0.011
33.2	Clinical remission	8 weeks	98	52	46	7	7	0	11	6	5	1	0.010
33.3	Clinical remission	8 weeks	102	56	46	11	11	0	11	6	5	3	0.029
34	Clinical response	8 weeks	102	51	51	45	32	13	14	4	10	9	0.088
35	Clinical remission	12 weeks	115	57	58	25	18	7	11	4	7	3	0.026
36	Clinical remission	52 weeks	297	145	152	96	60	36	84	28	56	10	0.034
37	Clinical response	7 days	104	53	51	74	44	30	0	0	0	4	0.038
38	Clinical responses or remission	3 months	70	34	36	35	29	6	10	6	4	17	0.243
39.1	Clinical response at both week 4 and week 6	6 weeks	31	15	16	5	5	0	0	0	0	1	0.032
39.2	Clinical response at both week 4	6 weeks	29	13	16	5	5	0	1	1	0	1	0.077

	and week 6											
40.1	Combined remission	52 weeks	204	103	101	97	58	39	81	37	44	4	0.020
40.2	Combined remission	52 weeks	170	86	84	82	49	33	0	37	44	3	0.018
40.3	Clinical remission	52 weeks	204	103	101	103	61	42	0	37	44	4	0.020
40.4	Clinical remission	52 weeks	194	99	95	99	59	40	0	37	44	3	0.015
41.1	Combined remission	24 weeks	212	107	105	89	53	36	41	19	22	2	0.009
41.2	Combined remission	24 weeks	204	103	101	89	53	36	0	19	22	2	0.010
41.3	Combined remission	24 weeks	194	99	95	89	53	36	0	19	22	2	0.010
42.1	Clinical response	8 weeks	66	35	31	36	25	11	26	12	14	4	0.061
42.2	Deep remission	8 weeks	56	33	23	14	12	2	26	12	14	1	0.018
42.3	Deep remission	48 weeks	51	24	27	6	6	0	47	25	22	2	0.039
43	Endoscopic remission	48 weeks	61	31	30	26	18	8	3	1	2	2	0.033
44	Remission	7 weeks	75	38	37	11	9	2	5	2	3	1	0.013
45	Composite	8 weeks	35	15	20	11	8	3	8	3	5	1	0.029

	endpoint											
46	Composite endpoint	8 weeks	81	41	40	14	11	3	20	9	11	1	0.012
47	Steroid-free remission	8 weeks	73	38	35	15	12	3	4	3	1	2	0.027

FI: fragility index; FQ: fragility quotient; FMT, fecal microbiota transplantation;

ID: exclusive code assigned to each study. When a study has multiple periods, such as induction or maintenance, or multiple parallel outcomes, or is a multi-arm study, it is divided into multiple trials. For example, study 3 was divided into trial 3.1 and 3.2.

Supplementary Table 6. Data of included trials for continuous fragile index and continuous fragility quotient

ID	Outcome	Follow up time	Sample size			Mean		Standard deviation		Discontinuation			CFI	CFQ
			Total	Interven-tional	Control	Interven-tional	Control	Interven-tional	Control	Total	Interven-tional	Control		
1.1	CDAI	12 weeks	122	61	61	-160.40	-36.20	192.185	192.185	1	1	0	14.2	0.1164
1.2	CDAI	12 weeks	124	63	61	-138.90	-36.20	192.380	192.380	2	2	0	12.0	0.0968
1.3	CDAI	12 weeks	120	59	61	-144.90	-36.20	192.506	192.506	3	3	0	12.2	0.1017
2	SES-CD score	8 weeks	180	83	97	-0.70	-3.00	4.970	5.260	null	null	null	16.8	0.0933
3.1	Mayo score	12 weeks	136	69	67	-3.06	-1.53	2.123	2.109	8	3	5	20.0	0.1471
3.2	Mayo score	12 weeks	134	67	67	-2.65	-1.53	2.113	2.109	6	1	5	13.2	0.0985
3.3	Mayo score	12 weeks	130	63	67	-2.80	-1.53	2.118	2.109	null	null	null	14.8	0.1138
4.1	Partial Mayo score	8 weeks	639	320	319	-2.60	-1.50	2.310	2.070	0	0	0	105.6	0.1653
4.2	Partial Mayo score	8 weeks	640	321	319	-2.90	-1.50	2.200	2.070	1	1	0	128.0	0.2000
4.3	CRP	8 weeks	631	315	316	-4.02	0.66	14.396	18.299	8	5	3	54.6	0.0865
4.4	CRP	8 weeks	636	320	316	-5.70	0.66	15.316	18.299	5	2	3	77.4	0.1217
4.5	FCP	8 weeks	585	296	289	-1211.28	17.92	4140.267	5873.016	54	24	30	36.0	0.0615
4.6	FCP	8 weeks	589	300	289	-1368.26	17.92	4755.094	5873.016	52	22	30	43.8	0.0744
4.7	FCP	8 weeks	600	306	294	-146.04	-40.14	356.182	329.986	41	16	25	60.6	0.1010
5.1	Partial Mayo score	54 weeks	63	32	31	-0.50	5.00	3.000	2.750	17	5	12	16.4	0.2603
5.2	Fecal	54 weeks	63	32	31	-0.04	0.76	1.475	1.450	17	5	12	2.4	0.0381

	lactoferrin													
5.3	FCP	54 weeks	63	32	31	0.00	0.54	0.725	0.850	17	5	12	4.8	0.0762
6.1	Modified Mayo Score	8 weeks	118	58	60	–2.9	–1.9	1.711	1.742	17	10	7	42.6	0.3610
6.2	Modified Mayo Score	8 weeks	114	54	60	–3.2	–1.9	1.832	1.742	17	10	7	42.8	0.3754
6.3	Modified Mayo Score	8 weeks	119	59	60	–3.1	–1.9	1.919	1.742	12	5	7	43.4	0.3647
7	Modified MCS	12 weeks	104	50	54	2.49	1.50	2.192	2.205	10	4	6	4.8	0.0462
8	Mayo Score	8 weeks	69	35	34	-2.80	-1.70	2.200	2.100	11	5	6	2.0	0.0290
9.1	CDAI	84 days	82	40	42	124.00	222.00	105.000	98.500	2	1	1	13.8	0.1683
9.2	CDAI	84 days	85	43	42	121.00	222.00	72.000	98.500	2	1	1	15.6	0.1835
10	Hs-CRP	16 weeks	73	36	37	-14.80	-0.10	26.400	12.000	17	7	10	12.0	0.1644
11.1	Mayo score	8 weeks	93	47	46	-2.00	-3.50	3.500	2.325	7	2	5	4.8	0.0516
11.2	Mayo score	8 weeks	102	56	46	-5.00	-3.50	3.250	2.325	11	6	5	8.6	0.0843
12	Mayo score	8 weeks	91	47	44	-4.05	-1.87	4.113	4.046	14	4	10	6.6	0.0725
13.1	CDAI	12 months	82	41	41	62.5	23.6	23.200	12.400	7	4	3	20.6	0.2512
13.2	HBI	12 months	82	41	41	3.4	1.2	1.200	0.580	7	4	3	22.2	0.2707
14	Simplified CDAI	12 months	22	11	11	1.70	0.5	1.400	0.500	5	3	2	1.0	0.0455

15.1	CDAI	12 months	42	21	21	−150.7	−63.0	99.480	114.700	3	2	1	3.8	0.0905
15.2	HBI	12 months	42	21	21	−6	−2	3.700	5.185	3	2	1	4.0	0.0952
15.3	SES-CD	12 months	40	21	19	−7	0	6.670	10.000	3	2	1	2.6	0.0650
16.1	ESR	48 weeks	61	31	30	10.10	20.50	2.800	5.300	7	1	2	15.8	0.2590
16.2	CRP	48 weeks	61	31	30	4.50	7.20	1.900	2.500	3	1	2	9.8	0.1607

CFI: continuous fragility index; CFQ: continuous fragility quotient; CDAI, Crohn's Disease Activity Index; SES-CD, Simple Endoscopic Score for CD; CRP, c-reactive protein; Hs-CRP, high-sensitivity C-reactive protein; HBI, Harvey-Bradshaw Index; FCP, fecal calprotectin; ESR, erythrocyte sedimentation rate.

Supplementary Table 7. Associations between trial characteristics and fragility index (including trials with FI=0)

Trial characteristics		FI		FQ	
Categorical variable	Trials for FI and FQ (%)	Median FI (IQR)	p value	Median FQ (IQR)	p value
**Total**	99 (100.0)	4 (2, 17)	\	0.0286 (0.0155, 0.0629)	\
**Disease**					
CD	42 (42.4)	6.5 (2, 20)	0.239	0.0304 (0.0171, 0.0601)	0.821
UC	57 (57.6)	4 (1, 15)		0.0280 (0.0136, 0.0711)	
**Treatment**					
FMT	11 (11.1)	1 (0, 2)	**<0.001**	0.0179 (0.0000, 0.0328)	0.062
Stem cell	10 (10.1)	2.5 (1.75, 4)		0.0186 (0.0102, 0.0329)	
Biologics	50 (50.5)	10 (3, 20)		0.0304 (0.0213, 0.0681)	
Small molecule drug	28 (28.3)	4 (1, 22.5)		0.0396 (0.0122, 0.0838)	
**Trial phase**					
2	32 (32.3)	1 (1, 3)	**<0.001**	0.0139 (0.0109, 0.0355)	**0.031**
3	55 (55.6)	11 (4, 23)		0.0340 (0.0202, 0.0670)	
1/2	3 (3.0)	6.3 (1, 17) [a]		0.1032 (0.0323, 0.2429) [a]	
2/3	1 (1.0)	2 (\) [b]		0.0328 (\) [b]	
Not mentioned	8 (8.1)	3 (0.25, 5.5)		0.0386 (0.0045, 0.0701)	
**Trial period**					
Induction	53 (53.5)	6 (1.5, 16.5)	**0.016**	0.0291 (0.0132, 0.0618)	0.052
Maintenance	25 (25.3)	11 (6, 25.5)		0.0569 (0.0257, 0.0985)	
**Type of analysis**					
ITT	66 (66.7)	7.5 (2, 17)	0.358	0.0289 (0.0153, 0.0679)	0.476

mITT	28 (28.3)	3 (1, 17.75)		0.0324 (0.0182, 0.0575)	
PP	5 (5.1)	3 (1.5, 10)		0.0155 (0.0094, 0.1303)	
**Blinding type**					
Double-blind	93 (93.9)	6 (2, 17)	0.004	0.0291 (0.0161, 0.0648)	0.068
Open-labelled	6 (6.1)	0.5 (0, 2.5)		0.0090 (0.0000, 0.0446)	
**Center**					
Multicenter	89 (89.9)	6 (2, 18.5)	0.110	0.0274 (0.0147, 0.0619)	0.497
Single-center	10 (10.1)	3 (0.75, 6.5)		0.0386 (0.0134, 0.0793)	
**Outcome type**					
Primary	74 (74.7)	4 (1.75, 16.25)	**0.006**	0.0290 (0.0155, 0.0709)	**0.011**
Coprimary	14 (14.1)	2.5 (1, 5)		0.0183 (0.0122, 0.0270)	
Secondary	10 (10.1)	20 (9.75, 23)		0.0529 (0.0317, 0.0675)	
Others	1 (1.0)	1 (\) [b]		0.0085 (\) [b]	
**Outcome definition**					
Clinical	69 (69.7)	8.0000 (2.5, 17)	**0.030**	0.0323 (0.0192, 0.0711)	**0.018**
Endoscopic	13 (13.1)	3.0000 (1, 23)		0.0411 (0.0164, 0.0689)	
Composite	17 (17.2)	2.0000 (1, 3.5)		0.0176 (0.0113, 0.0289)	
**Reported p-value**					
<0.05-0.01	36 (36.4)	1 (1, 2.75)	**<0.001**	0.0139 (0.0104, 0.0235)	**<0.001**
<0.01-0.001	18 (18.2)	6 (3, 8.5)		0.0262 (0.0206, 0.0383)	
<0.001	38 (38.4)	20 (11.75, 28)		0.0689 (0.0487, 0.0985)	
Not reported	7 (7.1)	1 (0, 4)		0.0085 (0.0000, 0.0325)	
**Recalculated p-value**					
>0.05	6 (6.1)	0 (0, 0)	**<0.001**	0.0000 (0.0000, 0.0000)	**<0.001**
<0.05-0.01	35 (35.4)	1 (1, 3)		0.0155 (0.0105, 0.0215)	
<0.01-0.001	23 (23.2)	7 (3, 10)		0.0317 (0.0244, 0.0411)	

<0.001	35 (35.4)	21 (16, 28)		0.0717 (0.0529, 0.1019)

**Continuous variable**	**Correlation Rs**	**p value**	**Correlation Rs**	**p value**
**Year of publication**	0.208	**0.039**	0.198	**0.049**
Impact factor	0.425	**<0.001**	0.159	0.116
Sample size				
Total	0.749	**<0.001**	0.305	**0.002**
Intervention	0.743	**<0.001**	0.296	**0.003**
Control	0.755	**<0.001**	0.313	**0.002**
**Number of discontinuations**				
Total	0.553	**<0.001**	0.271	**0.007**
Intervention	0.466	**<0.001**	0.194	0.056
Control	0.577	**<0.001**	0.308	**0.002**
Percentage of discontinuation	0.124	0.222	0.146	0.151
**Number of events**				
Total	0.722	**<0.001**	0.363	**<0.001**
Intervention	0.814	**<0.001**	0.467	**<0.001**
Control	0.555	**<0.001**	0.179	0.076
Percentage of events	0.274	**0.006**	0.269	**0.007**

Assessed using Wilcoxon rank-sum tests for binary variables, Kruskal－Wallis H tests for multiple categorical variables, and Spearman’ s correlation coefficient for continuous variables.

a Presented as mean (minimum maximum) as there are less than four data points.

b Present as unique data.

Percentage of discontinuation, total discontinuation divided by total sample size. Percentage of events, total number of events divided by total sample size.

FI, fragility index; FQ, fragility quotient; IQR, interquartile range.

REFERENCES

1. Abou Bakr A, Sarwar S, Aslam MN, Ghias M, Yousuf MN. To Determine the Frequency of Clinical Remission Induction with Versus Without Fecal Microbiota Transplant in Treatment of Active Ulcerative Colitis. *Annals of King Edward Medical University Lahore Pakistan.* Jan-Mar 2024;30(1):45-51.
2. Březina J, Bajer L, Wohl P, et al. Fecal Microbial Transplantation versus Mesalamine Enema for Treatment of Active Left-Sided Ulcerative Colitis-Results of a Randomized Controlled Trial. *J Clin Med.* Jun 22 2021;10(13).
3. Fang H, Fu L, Li X, et al. Long-term efficacy and safety of monotherapy with a single fresh fecal microbiota transplant for recurrent active ulcerative colitis: a prospective randomized pilot study. *Microb Cell Fact.* Jan 19 2021;20(1):18.
4. Hibi T, Motoye S, Ashida T, et al. Efficacy and safety of abrilumab, an α4β7 integrin inhibitor, in Japanese patients with moderate-to-severe ulcerative colitis: a phase II study. *Intestinal Research.* Jul 2019;17(3):375-386.
5. Atreya R, Peyrin-Biroulet L, Klymenko A, et al. Cobitolimod for moderate-to-severe, left-sided ulcerative colitis (CONDUCT): a phase 2b randomised, double-blind, placebo-controlled, dose-ranging induction trial. *Lancet Gastroenterology & Hepatology.* Dec 2020;5(12):1063-1075.

6. Sandborn WJ, Feagan BG, Wolf DC, et al. Ozanimod Induction and Maintenance Treatment for Ulcerative Colitis. *N Engl J Med.* May 5 2016;374(18):1754-1762.

7. Danese S, Vermeire S, Hellstern P, et al. Randomised trial and open-label extension study of an anti-interleukin-6 antibody in Crohn's disease (ANDANTE I and II). *Gut.* Jan 2019;68(1):40-48.

8. Rutgeerts P, Gasink C, Chan D, et al. Efficacy of Ustekinumab for Inducing Endoscopic Healing in Patients With Crohn's Disease. *Gastroenterology.* Oct 2018;155(4):1045-1058.

9. Vermeire S, Sandborn WJ, Danese S, et al. Anti-MAdCAM antibody (PF-00547659) for ulcerative colitis (TURANDOT): a phase 2, randomised, double-blind, placebo-controlled trial. *Lancet.* Jul 8 2017;390(10090):135-144.

10. Vermeire S, O'Byrne S, Keir M, et al. Etrolizumab as induction therapy for ulcerative colitis: a randomised, controlled, phase 2 trial. *Lancet.* Jul 26 2014;384(9940):309-318.

11. Feagan BG, Sandborn WJ, D'Haens G, et al. Induction therapy with the selective interleukin-23 inhibitor risankizumab in patients with moderate-to-severe Crohn's disease: a randomised, double-blind, placebo-controlled phase 2 study. *Lancet.* Apr 29 2017;389(10080):1699-1709.

12. D'Haens G, Panaccione R, Baert F, et al. Risankizumab as induction therapy for Crohn's disease: results from the phase 3 ADVANCE and MOTIVATE induction trials. *Lancet.* May 28 2022;399(10340):2015-2030.

13. Ferrante M, Panaccione R, Baert F, et al. Risankizumab as maintenance therapy for moderately to severely active Crohn's disease: results from the multicentre, randomised, double-blind, placebo-controlled, withdrawal phase 3 FORTIFY maintenance trial. *Lancet.* May 28 2022;399(10340):2031-2046.

14. Feagan BG, Sandborn WJ, Gasink C, et al. Ustekinumab as Induction and Maintenance Therapy for Crohn's Disease. *N Engl J Med.* Nov 17 2016;375(20):1946-1960.

15. Sands BE, Sandborn WJ, Panaccione R, et al. Ustekinumab as Induction and Maintenance Therapy for Ulcerative Colitis. *N Engl J Med.* Sep 26 2019;381(13):1201-1214.

16. Sands BE, Feagan BG, Rutgeerts P, et al. Effects of Vedolizumab Induction Therapy for Patients With Crohn's Disease in Whom Tumor Necrosis Factor Antagonist Treatment Failed. *Gastroenterology.* Sep 2014;147(3):618-+.

17. Sands BE, Chen J, Feagan BG, et al. Efficacy and Safety of MEDI2070, an Antibody Against Interleukin 23, in Patients With Moderate to Severe Crohn's Disease: A Phase 2a Study. *Gastroenterology.* Jul 2017;153(1):77-+.

18. Sandborn WJ, Ferrante M, Bhandari BR, et al. Efficacy and Safety of Mirikizumab in a Randomized Phase 2 Study of Patients With Ulcerative Colitis. *Gastroenterology.* Feb 2020;158(3):537-549.e510.

19. Peyrin-Biroulet L, Allegretti JR, Rubin DT, et al. Guselkumab in Patients With Moderately to Severely Active Ulcerative Colitis: QUASAR Phase 2b Induction Study. *Gastroenterology.* Dec 2023;165(6):1443-1457.

20. Sandborn WJ, Feagan BG, Marano C, et al. Subcutaneous Golimumab Maintains Clinical Response in Patients With Moderate-to-Severe Ulcerative Colitis. *Gastroenterology.* Jan 2014;146(1):96-+.

21. Colombel JF, Rutgeerts PJ, Sandborn WJ, et al. Adalimumab induces deep remission in patients with Crohn's disease. *Clin Gastroenterol Hepatol.* Mar 2014;12(3):414-422.e415.

22. Vermeire S, Danese S, Sandborn WJ, et al. Efficacy and Safety of the Anti-mucosal Addressin Cell Adhesion Molecule-1 Antibody Ontamalimab in Patients with Moderate-to-Severe Ulcerative Colitis or Crohn's Disease. *Journal of Crohns & Colitis.* 2023.

23. Allez M, Sands BE, Feagan BG, et al. A Phase 2b, Randomised, Double-blind, Placebo-controlled, Parallel-arm, Multicenter Study Evaluating the Safety and Efficacy of Tesnatilimab in Patients with Moderately to Severely Active Crohn's Disease. *J Crohns Colitis.* Aug 21 2023;17(8):1235-1251.

24. Suzuki Y, Motoya S, Hanai H, et al. Efficacy and safety of adalimumab in Japanese patients with moderately to severely active ulcerative colitis. *J Gastroenterol.* Feb 2014;49(2):283-294.

25. Hibi T, Imai Y, Senoo A, Ohta K, Ukyo Y. Efficacy and safety of golimumab 52-week maintenance therapy in Japanese patients with moderate to severely active ulcerative colitis: a phase 3, double-blind, randomized, placebo-controlled study-(PURSUIT-J study). *J Gastroenterol.* Oct 2017;52(10):1101-1111.

26. Motoya S, Watanabe K, Ogata H, et al. Vedolizumab in Japanese patients with ulcerative colitis: A Phase 3, randomized, double-blind, placebo-controlled study. *PLoS One.* 2019;14(2):e0212989.

27. Jiang X-L, Cui H-F, Gao J, Fan H. Low-dose Infliximab for Induction and Maintenance Treatment in Chinese Patients With Moderate to Severe Active Ulcerative Colitis. *Journal of Clinical Gastroenterology.* Aug 2015;49(7):582-588.

28. Vermeire S, Danese S, Zhou W, et al. Efficacy and safety of upadacitinib maintenance therapy for moderately to severely active ulcerative colitis in patients responding to 8 week induction therapy (U-ACHIEVE Maintenance): overall results from the randomised, placebo-controlled, double-blind, phase 3 maintenance study. *Lancet Gastroenterology & Hepatology.* Nov 2023;8(11):976-989.

29. Sands BE, Feagan BG, Peyrin-Biroulet L, et al. Phase 2 Trial of Anti-TL1A Monoclonal Antibody Tulisokibart for Ulcerative Colitis. *N*

Engl J Med. Sep 26 2024;391(12):1119-1129.

30. Louis E, Schreiber S, Panaccione R, et al. Risankizumab for Ulcerative Colitis: Two Randomized Clinical Trials. *Jama.* Sep 17 2024;332(11):881-897.

31. Loftus EV, Jr., Panés J, Lacerda AP, et al. Upadacitinib Induction and Maintenance Therapy for Crohn's Disease. *N Engl J Med.* May 25 2023;388(21):1966-1980.

32. Sandborn WJ, Feagan BG, D'Haens G, et al. Ozanimod as Induction and Maintenance Therapy for Ulcerative Colitis. *N Engl J Med.* Sep 30 2021;385(14):1280-1291.

33. Chen B, Zhong J, Li X, et al. Efficacy and Safety of Ivarmacitinib in Patients With Moderate-to-Severe, Active, Ulcerative Colitis: A Phase II Study. *Gastroenterology.* Dec 2022;163(6):1555-1568.

34. Danese S, Vermeire S, Zhou W, et al. Upadacitinib as induction and maintenance therapy for moderately to severely active ulcerative colitis: results from three phase 3, multicentre, double-blind, randomised trials. *Lancet (London, England).* 2022-06-04 2022;399(10341):2113-2128.

35. Sandborn WJ, Su C, Sands BE, et al. Tofacitinib as Induction and Maintenance Therapy for Ulcerative Colitis. *New England Journal of*

Medicine. May 4 2017;376(18):1723-1736.

36. Monteleone G, Neurath MF, Ardizzone S, et al. Mongersen, an oral SMAD7 antisense oligonucleotide, and Crohn's disease. *N Engl J Med.* Mar 19 2015;372(12):1104-1113.

37. Matsuoka K, Watanabe M, Ohmori T, et al. AJM300 (carotegrast methyl), an oral antagonist of α4-integrin, as induction therapy for patients with moderately active ulcerative colitis: a multicentre, randomised, double-blind, placebo-controlled, phase 3 study. *Lancet Gastroenterol Hepatol.* Jul 2022;7(7):648-657.

38. Sandborn WJ, Feagan BG, Loftus EV, Jr., et al. Efficacy and Safety of Upadacitinib in a Randomized Trial of Patients With Crohn's Disease. *Gastroenterology.* Jun 2020;158(8):2123-2138.e2128.

39. Sandborn WJ, Ghosh S, Panes J, et al. Efficacy of Upadacitinib in a Randomized Trial of Patients With Active Ulcerative Colitis. *Gastroenterology.* Jun 2020;158(8):2139-2149.e2114.

40. Yoshimura N, Watanabe M, Motoya S, et al. Safety and Efficacy of AJM300, an Oral Antagonist of α4 Integrin, in Induction Therapy for Patients With Active Ulcerative Colitis. *Gastroenterology.* Dec 2015;149(7):1775-+.

41. Danese S, Neurath MF, Kopoń A, et al. Effects of Apremilast, an Oral Inhibitor of Phosphodiesterase 4, in a Randomized Trial of Patients

With Active Ulcerative Colitis. *Clin Gastroenterol Hepatol.* Oct 2020;18(11):2526-2534.e2529.

42. Sands BE, D'Haens G, Panaccione R, et al. Ozanimod in Patients With Moderate to Severe Ulcerative Colitis Naive to Advanced Therapies. *Clin Gastroenterol Hepatol.* Oct 2024;22(10):2084-2095.e2084.

43. Singh A, Goyal MK, Midha V, et al. Tofacitinib in Acute Severe Ulcerative Colitis (TACOS): A Randomized Controlled Trial. *Am J Gastroenterol.* Jul 1 2024;119(7):1365-1372.

44. Hu J, Zhao G, Zhang L, et al. Safety and therapeutic effect of mesenchymal stem cell infusion on moderate to severe ulcerative colitis. *Exp Ther Med.* Nov 2016;12(5):2983-2989.

45. Melmed GY, Pandak WM, Casey K, et al. Human Placenta-derived Cells (PDA-001) for the Treatment of Moderate-to-severe Crohn's Disease: A Phase 1b/2a Study. *Inflamm Bowel Dis.* Aug 2015;21(8):1809-1816.

46. Panés J, García-Olmo D, Van Assche G, et al. Long-term Efficacy and Safety of Stem Cell Therapy (Cx601) for Complex Perianal Fistulas in Patients With Crohn's Disease. *Gastroenterology.* Apr 2018;154(5):1334-1342.e1334.

47. Panes J, Garcia-Olmo D, Van Assche G, et al. Expanded allogeneic adipose-derived mesenchymal stem cells (Cx601) for complex perianal fistulas in Crohn's disease: a phase 3 randomised, double-blind controlled trial. *Lancet.* Sep 24 2016;388(10051):1281-1290.

48. Kedia S, Virmani S, Vuyyuru SK, et al. Faecal microbiota transplantation with anti-inflammatory diet (FMT-AID) followed by anti-inflammatory diet alone is effective in inducing and maintaining remission over 1 year in mild to moderate ulcerative colitis: a randomised controlled trial. *Gut.* Dec 2022;71(12):2401-+.

49. Sood A, Mahajan R, Singh A, et al. Role of Faecal Microbiota Transplantation for Maintenance of Remission in Patients With Ulcerative Colitis: A Pilot Study. *J Crohns Colitis.* Sep 27 2019;13(10):1311-1317.

50. Moayyedi P, Surette MG, Kim PT, et al. Fecal Microbiota Transplantation Induces Remission in Patients With Active Ulcerative Colitis in a Randomized Controlled Trial. *Gastroenterology.* Jul 2015;149(1):102-109.e106.

51. Haifer C, Paramsothy S, Kaakoush NO, et al. Lyophilised oral faecal microbiota transplantation for ulcerative colitis (LOTUS): a randomised, double-blind, placebo-controlled trial. *Lancet Gastroenterology & Hepatology.* Feb 2022;7(2):141-151.

52. Paramsothy S, Kamm MA, Kaakoush NO, et al. Multidonor intensive faecal microbiota transplantation for active ulcerative colitis: a randomised placebo-controlled trial. *Lancet.* Mar 25 2017;389(10075):1218-1228.

53. Costello SP, Hughes PA, Waters O, et al. Effect of Fecal Microbiota Transplantation on 8-Week Remission in Patients With Ulcerative Colitis: A Randomized Clinical Trial. *Jama.* Jan 15 2019;321(2):156-164.

54. Sandborn WJ, D'Haens GR, Reinisch W, et al. Guselkumab for the Treatment of Crohn's Disease: Induction Results From the Phase 2 GALAXI-1 Study. *Gastroenterology.* May 2022;162(6):1650-1664.e1658.

55. Vermeire S, Sands BE, Tilg H, et al. ABX464 (obefazimod) for moderate-to-severe, active ulcerative colitis: a phase 2b, double-blind, randomised, placebo-controlled induction trial and 48 week, open-label extension. *Lancet Gastroenterology & Hepatology.* Nov 2022;7(11):1024-1035.

56. Sandborn WJ, Peyrin-Biroulet L, Zhang J, et al. Efficacy and Safety of Etrasimod in a Phase 2 Randomized Trial of Patients With Ulcerative Colitis. *Gastroenterology.* Feb 2020;158(3):550-561.

57. Zhang J, Lv S, Liu X, Song B, Shi L. Umbilical Cord Mesenchymal Stem Cell Treatment for Crohn's Disease: A Randomized Controlled Clinical Trial. *Gut Liver.* Jan 15 2018;12(1):73-78.

58. Zhou C, Li M, Zhang Y, et al. Autologous adipose-derived stem cells for the treatment of Crohn's fistula-in-ano: an open-label, controlled trial. *Stem Cell Research & Therapy.* Mar 17 2020;11(1).

59. Hawkey CJ, Allez M, Clark MM, et al. Autologous Hematopoetic Stem Cell Transplantation for Refractory Crohn Disease: A Randomized Clinical Trial. *Jama.* Dec 15 2015;314(23):2524-2534.
